# Layered Anion‐Mixed Oxycompounds: Synthesis, Properties, and Applications

**DOI:** 10.1002/advs.202500477

**Published:** 2025-02-07

**Authors:** Yange Luan, Yumin Li, Zhong Li, Bao Yue Zhang, Jian Zhen Ou

**Affiliations:** ^1^ School of Engineering RMIT University Melbourne 3000 Australia; ^2^ Key Laboratory of Advanced Technologies of Materials Ministry of Education School of Materials Science and Engineering Southwest Jiaotong University Chengdu 610031 China

**Keywords:** layered anion‐mixed oxycompound, oxychalcognides, oxyhalides, oxynitrides, oxypnictides

## Abstract

Layered anion‐mixed oxycompounds have emerged as pivotal materials across diverse technological domains encompassing electronics, optics, sensing, catalysis, and energy applications. Capitalizing on the unique properties imparted by the additional anion, these compounds exhibit exceptional characteristics including ultra‐large charge carrier mobility, giant second‐harmonic generation, visible‐light‐driven photocatalysis, and outstanding thermoelectricity. This article aims to provide a comprehensive summary of layered anion‐mixed oxychalcogenides, oxyhalides, oxynitrides, and oxypnictides. Organized by chemical composition and crystal structures, the classification of these oxycompounds precedes an in‐depth exploration of various synthesis methodologies. Subsequently, their properties are elucidated in electronics, optics, magnetics, and ferroelectrics, contextualizing their utility in electronic, optical, and catalytic applications. The review culminates in a critical assessment of extant challenges and opportunities within this realm. Furthermore, insights are proffered into the future trajectory of research, underpinning the significance of advancing novel 2D multi‐anion oxygenated compounds and their attendant applications.

## Graphical abstract

This article offers a thorough review covering the entire spectrum of layered anion‐mixed oxy‐compound materials, specifically highlighting their crystal structures, synthesis techniques, properties, and applications. The summary provides a picture of the field's current advancements and offers perspectives on future directions.

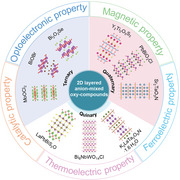

## 1 Introduction

Recent years have witnessed significant advancements in the study of layered materials, driven by the strategic cleavage of van der Waals (vdW) bonds between monolayers and innovative bottom‐up growth techniques. This technological leap has ushered in a transformative era of applications centered around 2D materials across a spectrum of scientific disciplines. Within this landscape, an emerging class of mixed‐anion oxycompounds, characterized by the incorporation of additional anionic species alongside oxygen, has garnered considerable attention. These compounds exhibit a distinctive layer stacking characteristic, upheld by interlayer electrostatic or vdW forces.^[^ ^1^ ^]^ The field of anion engineering in oxide‐based compounds demonstrates that the incorporation of different anions can alter the physical properties of materials. By introducing additional anions with varying sizes, electronegativities, and charges, materials such as oxychalcogenides, oxyhalides, oxynitrides, and oxypnictides can exhibit unique or enhanced properties that are not observed in their oxide counterparts.^[^ ^2^ ^]^ This is due to the diverse polarizabilities of the anions, which allow for more complex atomic structures and interactions. For example, layered anion‐mixed materials, such as bismuth oxyhalides and oxysulfides, exhibit narrower bandgaps compared to their oxide counterparts, allowing them to harvest visible photons for efficient overall water splitting,^[^ ^1^, ^3^ ^]^ dye degradation,^[^ ^4^ ^]^ nitrogen fixation,^[^ ^5^ ^]^ and carbon dioxide reduction.^[^ ^6^ ^]^ Additionally, certain layered anion‐mixed oxycompounds exhibit remarkable charge carrier mobility. For example, 2D Bi_2_O_2_Se, with its ultralow electron mass, showcases extraordinary Hall mobility of up to 28 900 cm^2^ V^−1^ s^−1^ at low temperatures, alongside a current on/off ratio of 10^6^ and nearly ideal subthreshold swing of 65 mV dec^−1^.^[^ ^7^ ^]^ Similarly, low‐symmetry oxycompounds like layered NbOI_2_ exhibit nonlinear optical responses and tunable second‐harmonic generation (SHG),^[^ ^8^ ^]^ surpassing the performance of the majority of 2D nonlinear materials.^[^ ^9^ ^]^ Moreover, these materials exhibit high magnetic susceptibility,^[^ ^1^, ^10^ ^]^ and anisotropic ferroelectricity,^[^ ^11^ ^]^ underscoring their multifaceted utility. With the introduction of more cations in anion‐mixed oxycompounds, their crystal structures tend to form segregated layers, bringing unique physical properties.^[^ ^12^ ^]^ For example, quaternary oxychalcogenides crystallize with alternating oxide and covalent chalcogenide layers. The covalent nature of chalcogenide layers facilitates high‐mobility semiconducting behavior, while the oxide blocks contribute to low thermal conductivity. This combination of ionic oxide anions and more covalent chalcogenide anions results in a unique structural chemistry, offering a promising new avenue of research in modern materials science.^[^ ^13^ ^]^ The naturally layered anion‐mixed oxycompounds are highly appealing for 2D‐enabled nanotechnology. These structures are predominantly interconnected through ionic bonds, while the rest are linked by vdW force. Such a layered structure facilitates the isolation of bulk parental materials into mono‐ or few‐layers. This characteristic offers significant advantages for the development of miniaturized electronic devices in contemporary high‐tech applications.

**Figure 1:**
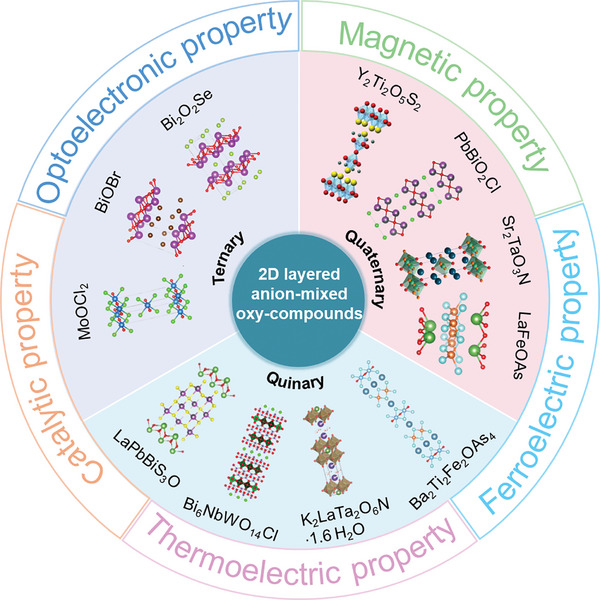
Overview of layered mixed‐anion oxycompounds and their properties.

In recent years, the growing research interest in layered anion‐mixed oxycompounds has spurred the publication of several insightful review articles, covering topics such as oxysulfides,^[^ ^14^ ^]^ solar energy applications,^[^ ^15^ ^]^ and progress for specific material like Bi_2_O_2_Se.^[^ ^16^ ^]^ Despite these commendable efforts, a comprehensive review encompassing the entirety of research progress in this field remains elusive. This article aims to address this gap by providing a thorough and systematic overview of the composition, synthesis methods, properties, and applications of recently reported layered oxycompounds. It is important to note that current research on this class of materials is primarily focused on Bi‐based compounds. This focus is driven by bismuth's high oxygen affinity, enabling stable Bi─O bonds and Bi_2_O_2_ layer formation, along with its excellent thermodynamic stability and controllable synthesis methods. As shown in **Figure** **1**, the organization of this paper is as follows. The materials are categorized based on the number of anions and types of cations, encompassing ternary, quaternary, and quinary oxychalcogenides, oxyhalides, oxynitrides, and oxypnictides respectively. Subsequently, their optical, electronic, thermoelectric, magnetic, and ferroelectric properties are elucidated, providing a comprehensive understanding of their multifaceted characteristics. Furthermore, we delve into their catalytic applications across various processes including water splitting, dye degradation, nitrogen fixation, and CO_2_ reduction, highlighting their potential contributions to sustainable technology solutions. The review also accentuates their electronic and optical applications, spanning field‐effect transistors, photodetectors, solar cells, thermoelectric, and optical sensor devices, underscoring their versatility across diverse technological domains. Finally, the conclusion and outlook section discuss the remaining challenges in the research landscape, while also emphasizing the burgeoning enthusiasm among investigators for the exploration of layered anion‐mixed oxycompounds. As this field continues to evolve, it promises to illuminate the path toward unlocking the mysteries inherent in nano‐enabled technologies.

## 2 Composition and Crystal Structures

Crystal structure and composition are intricately linked to the properties and functionalities of materials. In this chapter, we present a detailed overview of reported layered oxycompounds and their crystal structures, categorizing them based on the varying numbers of metal cations and distinct anion types. To improve clarity, we have added an explanation of the symbols and terms used throughout this review. The layered anion‐mixed oxycompounds can be classified into ternary, quaternary, and quinary compounds, represented by general formulas of AOCh and AOX for ternary; ABOCh, ABOX, ABON, and ABOPn for quaternary; ABCOCh, ABCOX, ABCON, and ABCOPn for quinary. In these formulas, A, B, and C denote metal cations, Ch represents chalcogens (S, Se, Te); X represents halogens (Cl, Br, I), N represents nitrogen, and Pn represents pnictogen (P, As). The general formula here contains only the elemental composition of compounds without considering the stoichiometric ratio. The atomic ratio of different elements refers to the specific composite of an anion‐mixed oxycompound.

The crystal structures and lattice parameters of reported layered oxycompounds with mixed‐anion are summarized in **Table** **1**.

**Table 1:** The crystal structure and basic parameters of layered anion‐mixed oxycompounds.

Materials	Crystal structure^a)^	Crystal system and Space group	Lattice parameters (Å)	Type of interlayer force	Ref.
Bi_2_O_2_S	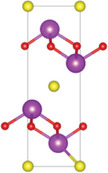	Orthorhombic *Pnnm*	*a* = 3.874 *b* = 3.84 *c* = 11.92	Electrostatic forces	[17]
Bi_2_O_2_Se	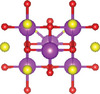	Tetragonal *I*4*/mmm*	*a* = 3.88 *b* = 3.88 *c* = 12.16	Electrostatic forces	[7]
Bi_2_O_2_Te		Tetragonal *I*4*/mmm*	*a* = 3.98 *b* = 3.98 *c* = 12.70	Electrostatic forces	[18]
Bi_4_O_4_S_3_	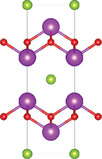	Tetragonal *I*4*/mmm*	*a* = 3.9697(2) *b* = 3.9697(2) *c* = 41.3520(1)	–	[19]
Bi_9_O_7.5_S_6_	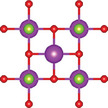	Trigonal *R* $\bar{3}$ *m*	*a* = 4.0685(7) *b* = 4.0685(7) *c* = 31.029(5)	–	[20]
BiOCl	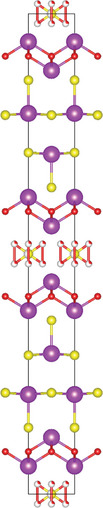	Tetragonal *P*4*/nmm*	*a* = 3.891 *b* = 3.891 *c* = 7.369	vdW forces	[21]
BiOBr		Tetragonal *P*4*/nmm*	*a* = 3.927 *b* = 3.927 *c* = 8.101	vdW forces	[22]
BiOI		Tetragonal *P*4*/nmm*	*a* = 3.994 *b* = 3.994 *c* = 9.149	vdW forces	[23]
VOCl	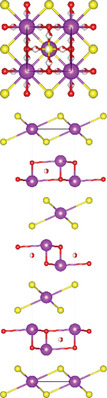	Orthorhombic *Pmmn*	*a* = 3.78 *b* = 3.30 *c* = 7.91	vdW forces	[24]
FeOCl		Orthorhombic *Pmmn*	*a* = 3.78 *b* = 3.30 *c* = 7.92	vdW forces	[1d]
YbOCl	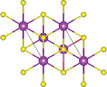	Trigonal R $\bar{3}$ *m*	*a* = 3.726 *b* = 3.726 *c* = 27.830	vdW forces	[25]
MoOCl_2_	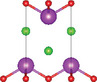	Monoclinic *C*2/*m*	–	vdW forces	[26]
NbOI_2_		Monoclinic *C*2	*a* = 15.18 *b* = 3.92 *c* = 7.52 β = 105.5°	vdW forces	[27]
BiCuSeO	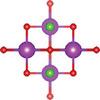	Tetragonal *P*4*/nmm*	a = 3.9273 b = 3.9273 c = 8.9293	Electrostatic forces	[28]
Y_2_Ti_2_O_5_S_2_	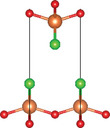	Tetragonal *I*4/*mmm*	a = 3.80 b = 3.80 c = 23.09	Electrostatic forces	[3a]
PbBiO_2_Cl	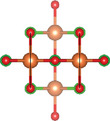	Tetragonal *I*4/*mmm*	a = 6.956 b = 6.956 c = 5.654	Electrostatic forces	[29]
Bi_4_NbO_8_Cl	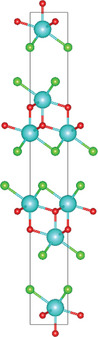	Tetragonal *P*4*/mmm*	*a* ∼ 3.9 *b* ∼ 3.9 *c* ∼ 14.4	Electrostatic forces	[3b]
Bi_2_GdO_4_Cl	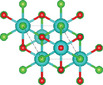	Tetragonal *P*4*/mmm*	–	Electrostatic forces	[3c]
SrBiO_2_Cl	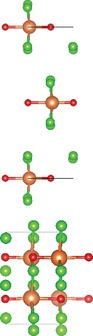	Orthorhombic *Cmcm*	–	Electrostatic forces	[3c]
Sr_2_TaO_3_N	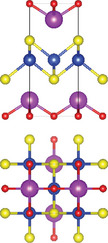	Tetragonal *I*4/*mmm*	*a* = 4.0448(2) *b* = 4.0448(2) *c* = 12.6002(7)	Electrostatic forces	[30]
LaFeOAs	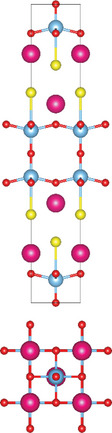	Tetragonal *P*4/*nmm*	*a = 4.03552(8)* *b = 4.03552(8)* *c = 8.7393(2)*	Electrostatic forces	[31]
Sr_2_Cr_3_As_2_O_2_	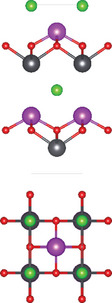	Tetragonal *I*4/*mmm*	*a = 4.0079(1)* *b = 4.0079(1)* *c = 18.8298(3)*	–	[32]
LaPbBiS_3_O	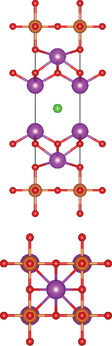	Tetragonal *P*4*/nmm*	*a* = 4.0982(1) *b* = 4.0982(1) *c* = 19.7754(6)	Electrostatic forces	[33]
Ba_2_CoO_2_Ag_2_Se_2_	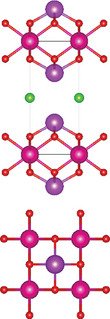	Tetragonal *I*4/*mmm*	*a* = 4.22323(7) *b* = 4.22323(7) *c* = 20.0358(4)	Electrostatic forces	[34]
Ba_2_MnO_2_Ag_2_Se_2_		Tetragonal *I*4/*mmm*	*a* = 4.26514(5) *b* = 4.26514(5) *c* = 19.7184(2)	Electrostatic forces	[34]
Bi_6_NbWO_14_Cl	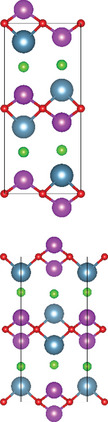	Orthorhombic *Fmmm*	*a* = 5.47826(5) *b* = 5.44114(5) *c* = 45.2874(4)	Electrostatic forces	[35]
NaLaTiO_4–x_N_y_	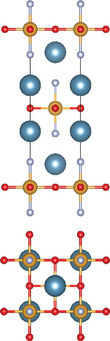	Tetragonal *P*4*/nmm*	*a* = 3.7830(1) *b* = 3.7830(1) *c* = 13.0567(4)	–	[36]
Li_2_LaTa_2_O_6_N	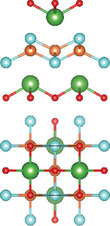	Tetragonal *I*4/*mmm*	*a* = 3.9533(4) *b* = 3.9533(4) *c* = 18.452(3)	Electrostatic forces	[37]
K_2_LaTa_2_O_6_N⋅1.6 H_2_O	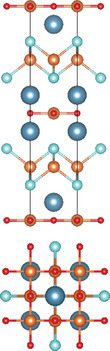	Tetragonal *P*4*/mmm*	*a* = 3.96116(5) *b* = 3.96116(5) *c* = 12.8565(3)	Electrostatic forces	[1f]
Ba_2_Ti_2_Fe_2_As_4_O	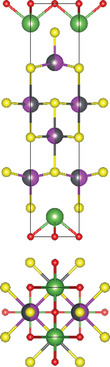	Tetragonal *I*4/*mmm*	*a = 4.033* *b = 4.033* *c = 27.38*	–	[38]

^a)^ Please refer to the corresponding references for detailed atomic models.

### 2.1 Ternary Anion‐Mixed Oxycompounds

#### 2.1.1 Ternary Oxychalcogenides (AOCh, A = Metal; Ch = S, Se, Te)

Ternary oxychalcogenides are typically layered compounds with the general formula of AOCh, where A is a metal element and Ch is a chalcogen. The types of crystal structures depend on the stoichiometric compositions of the compounds. If the stoichiometric ratio of metal (or oxygen) and chalcogenide is 2:1 (i.e. A_2_O_2_Ch), the covalent bonding between metal and oxygen atoms form metal oxide layers (A_2_O_2_), which are separated by chalcogenide layers through electrostatic interactions. Cheng et al. proposed a Zipper‐like structure, known as the “zipper model,” to illustrate layered Bi_2_O_2_Ch. The Zipper‐like structure specifically refers to the phenomenon where the 50% of Ch on the top surface of the bottom monolayer and the bottom surface of the top monolayer merge to form a single Ch layer when Bi_2_O_2_Ch is in a bilayer configuration. Using the High‐angle annular dark‐field scanning transmission electron microscopy (HAADF‐STEM), a 50% Ch‐passivation surface of Bi_2_O_2_Ch was observed.^[^ ^1a^ ^]^ However, there is still a small discrepancy between Bi_2_O_2_S and Bi_2_O_2_Se/ Bi_2_O_2_Te in terms of crystal structures. Bi_2_O_2_Se, and Bi_2_O_2_Te exhibit highly symmetrical tetragonal structures with the *I*4/mmm space group. Their crystal structures consist of planar covalently bonded oxide layers (Bi_2_O_2_) sandwiched by Se or Te layers.^[^ ^18^, ^39^ ^]^ On the other hand, Bi_2_O_2_S crystallizes in the orthorhombic *Pnnm* space group due to the slight distortion of fluorite‐like (Bi_2_O_2_)^2+^ slabs.^[^ ^17^ ^]^ When the stoichiometric ratio of metal (or oxygen) and chalcogenide is less than 2:1, compounds can be illustrated by a three‐layer coordination (i.e. metal oxide layer/metal chalcogenide layer / and chalcogenide oxide layer) stacked via electrostatic interactions.^[^ ^7^, ^17^, ^40^ ^]^ For example, Bi_4_O_4_S_3_ shows a layered tetragonal structure in *I*4*/mmm* space group including Bi_2_S_4_ (rock salt‐type), Bi_2_O_2_ (fluorite‐type), and SO_4_ layers.^[^ ^19^ ^]^ The further decrease of stoichiometric ratio of oxygen and chalcogenide (i.e. Bi_9_O_7.5_S_6_) transforms the three‐layer coordination to two‐layer coordination consisting solely of [Bi_2_O_2_] and [BiS_2_] layers.^[^ ^20^ ^]^

#### 2.1.2 Ternary Oxyhalides (AOX, A = Metal; X = Cl, Br, I)

Ternary oxyhalides, with the general formula of AOX, are typically composed of repeated sextuple layers (X‐A‐O‐O‐A‐X, A: metal atom, O: oxygen atom, and X: halide atom), in which the two halide layers clamp the metal‐O bilayers via covalent bonds or electrostatic interactions. These sextuple layers are stacked together via vdW forces along the *c* direction to form the layered ternary oxyhalides, which are distinctly different from the ternary oxychalcogenides. There are several crystal types of ternary oxyhalides including AOX (e.g. BiOX, YbOCl, and VOCl) and AOX_2_, according to their out‐of‐nuclear electronic layouts of the metal cations and different elemental ratios. For example, BiOBr exhibits a tetragonal PbFCl‐type layered structure with space group *P4/nmm*. The structure consists of double [Br]^−^ layers encapsulated between [Bi_2_O_2_]^2+^ layers.^[^ ^22^ ^]^ The in‐plane atoms in [Bi_2_O_2_]^2+^ layers are interconnected by covalent bonds, while the halogen ions in [Br]^−^ layers are bonded by electrostatic forces. In contrast to BiOX, VOCl shows an orthorhombic symmetry crystal structure with a space group *Pmmn* (lattice parameter: *a* = 3.78 Å, *b* = 3.30 Å and *c* = 7.91 Å). Such structure contains a buckled V‐O bilayer sandwiched by two Cl layers governed by covalent bonds.^[^ ^24^ ^]^ YbOCl is a new representative layered AOX material with a trigonal space group, which consists of repeating sextuple layers of Cl‐Yb‐O‐O‐Yb‐Cl.^[^ ^25^ ^]^ Through the top view of the YbOCl crystal structure, a single [Yb_4_O] tetrahedral unit can be seen with the formation of skeleton by Yb atoms, while the O atom occupies its interstice. In addition to AOX, AOX_2_ can be observed in the V‐, Ta‐, Nb‐, Mo‐, Ru‐, and Os‐based compounds. In their crystal structures, the AO_2_X_4_ octahedra are corner‐sharing (via O atoms) along the *a‐*axis, while edge‐sharing (via X atoms) along the *b*‐axis.^[^ ^26^, ^27^, ^41^ ^]^

### 2.2 Quaternary Anion‐Mixed Oxycompounds

#### 2.2.1 Quaternary Oxychalcogenides (ABOCh, A, B = Metal; Ch = S, Se, Te)

Bication oxychalcogenides (ABOCh, where A, B = Metal; Ch = S, Se, and Te) consist of two metallic elements, oxygen, and chalcogen. The introduction of an additional metal element induces changes in its crystal structures compared to the single‐cation counterpart.^[^ ^42^ ^]^ In bication oxychalcogenides, two metal cations typically exhibit significantly different oxophilicity and chalcophilicity, leading to the formation of alternating stacks of oxide and chalcogenide layers. The covalent nature of the chalcogenide layer enhances high‐mobility semiconduction, while the oxide layer contributes to low thermal conductivity.^[^ ^43^ ^]^ The presence of both ionic oxide anions and more covalent chalcogenide anions creates a distinct structural chemistry, which presents new opportunities in the field of material science.^[^ ^44^ ^]^ Moreover, quaternary oxychalcogenides can possess various types of crystal structures depending on their stoichiometric ratio, further expanding their potential applications. For example, a Cu‐based quaternary oxychalcogenide has a chemical formula of ACuOCh,^[^ ^42a^ ^]^ where A represents Bi, Y, and trivalent lanthanide ions. CuCh and AO layers are stacked via electrostatic interactions. Another crystal structure of interest falls in Ti‐based quaternary oxychalcogenides with an atomic ratio of A:Ti:O:S of 2:2:5:2 (A_2_Ti_2_O_5_S_2_, A = lanthanide).^[^ ^3a^ ^]^ Ti atom is surrounded by five oxygen atoms and one sulfur atom, forming double octahedra layers. Atoms A are located at both terminals of the double octahedra, connected by sulfur atoms. These double octahedra layers, surrounded by A atoms at their upper and lower ends, are sandwiched by individual octahedra layers via electrostatic interactions. Y_2_Ti_2_O_5_S_2_, which crystallizes in the *I*4/mmm space group, comprises a Ruddlesden‐Popper type, in which corner‐sharing of Ti_2_O_5_ octahedra constructed perovskite slabs are separated by rock salt slabs [Y_2_S_2_]^2+^.

#### 2.2.2 Quaternary Oxyhalides (ABOX, A, B = Metal; X = Cl, Br, I)

Bication oxyhalides (ABOX, A, B = Metal; X = Cl, Br, I) consist of two metal elements, one oxygen, and one halide element. Bi‐based bication oxyhalides have been extensively studied so far within this category. The difference in their crystal structures is mainly reflected by the coordination of the metal oxide layer due to various arrangements of the outermost electrons in the electron cloud surrounding the metal atomic nucleus. Here, four typical Bi‐based crystal structures are summarized. 1) PbBiO_2_X (X = Cl, Br) crystallizes in the *I*4/*mmm* space group with the tetragonal crystal structure.^[^ ^29^, ^45^ ^]^ The tetragonal [PbBiO_2_] layers are sandwiched by the [X] layers along the *c*‐axis. 2) Bi_4_MO_8_Cl (M = Nb, Ta) is a single layer Sillén‐Aurivillius perovskite consisting of MO_4_ perovskite blocks which are separated by (Bi_2_O_2_)_2_Cl blocks.^[^ ^3^, ^46^ ^]^ 3) Bi_2_GdO_4_X (X = Cl, Br) has a space group of *P*4/*mmm* formed by the stacking of [Bi_2_GdO_4_]^+^ and [X]^−^ layers. The [M_3_O_4_]^+^ layer is constructed from double 2[M_2_O_2_]^2+^ layers that are joined together by removing one M^3+^ layer. The Gd ion is coordinated by the eight oxygens at the vertices of a pseudocube.^[^ ^3c^ ^]^ 4) SrBiO_2_X (X = Cl, Br, I) features an alternate arrangement of [SrBiO_2_]^+^ and [X]^−^ layers and is closely related to BiOX (*P*4/*mmm*) containing [Bi_2_O_2_]^2+^ and [X_2_]^2−^ layers. This structural relationship arises from the replacement of one [X]^−^ layer with Sr due to aliovalent Sr for Bi substitution.

#### 2.2.3 Quaternary Oxynitrides and Oxypnictides (ABON and ABOPn, A, B = Metal, Pn = P, As)

Layered bication oxynitrides (ABON) are usually derived from non‐layered perovskite metal oxynitrides ABO_x_N_3‐x_ (A = Ca, Sr, Ba, La and Eu; B = Nb, Ta and Zr). By inserting the AO layers in the perovskite structure of ABO_x_N_3‐x_, perovskite metal oxynitrides with the formula of ABON are developed. For instance, SrTaO_2_N has an orthorhombic crystal structure and N atoms are separated into the perovskite lattice layer, where inequivalent O and O_0.5_N_0.5_ anion sites appear in the *ab* plane.^[^ ^30^ ^]^ Extra SrO layer insertion in perovskite SrTaO_2_N and disconnection of TaO_4_N_2_ octahedron together facilitate the formation of a Ruddlesden‐Popper compound – Sr_2_TaO_3_N. The general formula of Ruddlesden‐Popper oxynitride is written in A_2_[A_n−1_B_n_(O,N)_3n+1_], where A and B are metal cations and n = integer. Ca_3_Nb_2_N_2_O_5_ is another example of an (*n* = 2) Ruddlesden‐Popper perovskite oxynitride. Its crystal structure features alternating rock salt layers composed of Ca‐O, situated between two perovskite units. These CaNbO_2_N perovskite blocks stack along the pseudocubic [001] direction. Bication oxyphosphides and oxyarsenides have two types of crystal structures, A_2_B_3_Pn_2_O_2_ (A = alkaline metals: Sr/Ba; B = transition metals: Mn/Cr, Pn = As) and LaBOPn (B = Mn/Fe/Co/Ni/Zn, Pn = P/As). In the A_2_B_3_Pn_2_O_2_ structure (also known as A_2_Mn_3_Pn_2_O_2_), the (A_2_BO_2_) layers containing square sheets of (BO_2_), are separated by anti‐PbO‐type (BPn) layers with transition metal ions located in edge‐shared (BPn_4_) tetrahedra.^[^ ^47^ ^]^ This structure is also adopted by some of quinary oxychalcogenides, such as Ba_2_CoO_2_Ag_2_Se_2_. On the other hand, LaBOPn shares ZrCuSiAs structure, characterized by alternating (LaO) and (BPn) layers stacked along the *c*‐axis.^[^ ^7^ ^]^

### 2.3 Quinary Anion‐Mixed Oxycompounds

#### 2.3.1 Quinary Oxychalcogenides (ABCOCh, A, B, C = Metal; Ch = S, Se, Te)

Quinary oxychalcogenides exhibit two distinct crystal structures depending on the types of metal atoms present.^[^ ^33^, ^34^, ^48^ ^]^ When two (metal B and C) of the three metal atoms have similar atomic numbers (e.g. M = Pb and Bi), the resulting crystal structure is composed of [A_2_O_2_] and [M_4_Te_6_] layers. For example, LaPbBiS_3_O is a quinary oxychalcogenide with a tetragonal *P*4/*nmm* space group, composed of stacked [M_4_S_6_] layers and fluorite‐type [La_2_O_2_] layers.^[^ ^33^ ^]^ The second type of crystal structure involves three metal atoms which belong to different columns in the periodic table. Typically, one of metal atoms in the first subfamily (A = Cu and Ag) combines with chalcogenides to form [A_2_Ch_2_] layers. Part of B atoms (B = Sr and Ba) is combined with oxygen atoms, while the rest exists alone in the interlayer gap to separate the oxide and chalcogenide layers. The lattice structure of the metal oxide layer is determined by the C metal atom. For example, Sr_3_Cu_2_Fe_2_O_5_S_2_ has a body‐centered tetragonal crystal structure, where the Cu_2_S_2_ layer is stacked with the iron perovskite oxide layer (FeO_2_) (SrO) (FeO_2_) separated by Sr.^[^ ^48a^ ^]^ Sr_2_CuAO_3_S (A = Cr, Fe) is composed of square‐pyramidal oxide layers, specifically ([(AO_3_)(SrO)(SrO)(AO_3_)]), which are stacked with Cu_2_S_2_ layers, In this structure, Sr atoms are located in the interlayer gap. A_2_BO_2_C_2_Se_2_ (A = Sr/Ba, B = Co/Mn/Zn, C = Cu/Ag) also exhibits A_2_Mn_3_Pn_2_O_2_^–^ type structure (space group *I*4/mmm), which was first observed in quaternary oxypnictides. This structure is characterized by stacking (BO_2_) layers with anti‐fluorite‐type (C_2_Se_2_) layers along the *c‐* axis, separated by A atoms.^[^ ^34^ ^]^

#### 2.3.2 Quinary Oxyhalides (ABCOX, A, B, C = Metal; X = Cl, Br, I)

The study of quinary oxyhalides has been limited due to the complexity of their construction and the challenging experimental conditions. Currently, there is only one known quinary oxyhalide – Bi_6_NbWO_14_Cl sharing a similar crystal structure with bication oxyhalides, where halogen layers separate metal oxide layers. Bi_6_NbWO_14_Cl adopts an orthorhombic structure consisting of perovskite [(Nb_0.5_W_0.5_)O_4_]^2.5−^ layers, which are sandwiched by [(Bi_2_O_2_)_2_Cl]^3+^ and [Bi_2_O_2_]^2+^ blocks.^[^ ^35^ ^]^

#### 2.3.3. Quinary Oxynitrides and Oxypnictides (ABCON and ABCOPn, A, B, C = Metal, Pn = P, As)

Layered quinary oxynitrides exhibit both the intrinsic and doping forms. For intrinsic compounds, layered perovskite oxynitride Li_2_LaTa_2_O_6_N has a tetragonal (*I*4/*mmm*) structure, which consists of double‐layered [LaTa_2_O_6_N]^2−^ perovskite blocks which are separated by Li^+^ cations.^[^ ^37^ ^]^ On the other hand, hydrated K_2_LaTa_2_O_6_N exhibits an (*n* = 2) Ruddlesden‐Popper‐type perovskite structure, where corner‐sharing TaO_6‐x_N_x_ octahedral units form double‐layer perovskite blocks stacked along the *c‐*axis. K^+^ ions and the oxygen atoms of water molecules are located in the interlayer gap.^[^ ^1f^ ^]^ For doping counterparts, they are realized by introducing nitrogen dopants into parental metal oxide layers. NaLaTiO_4‐x_N_y_ is an example of doped oxynitrides, where La^3+^ and Na^+^ cations accommodate both sides of off‐centering Ti(O, N)_6_ octahedrons.^[^ ^36^ ^]^ However, doped oxynitrides often contain oxygen vacancies due to the mismatch in charge between oxide (O^2−^) and nitride anions and (N^3−^), as well as the reduction of Ti^4+^ to Ti^3+^. For instance, in NaLaTiO_4_, an oxygen vacancy is produced when O^2−^ is replaced by N^3−^ to maintain the overall charge balance. Except for quinary oxynitrides, there is one quinary oxyarsenide (Ba_2_Ti_2_Fe_2_As_4_O) reported.^[^ ^38^ ^]^ Its crystal structure is a combination of BaFe_2_As_2_ and BaTi_2_As_2_O, where blocks of Fe_2_As_2_, Ba, and Ti_2_As_2_O are stacked along the *c*‐axis of the tetragonal cell with *I*4/*mmm* space group

## 3 Synthetic Methods

Layered anion‐mixed oxycompounds are conventionally synthesized using high‐temperature solid‐state reactions. However, recent studies have revealed various synthesis methodologies to achieve precise control over the composition, morphology, and structural properties of these compounds. These methodologies include the polymerized complex method, chemical vapor‐based methods, hydrothermal and solvothermal techniques, among others. Each method was selected for its unique character to produce materials with specific properties, such as enhanced electrical conductivity, mechanical strength, or tailored catalytic activity. Additionally, some methods allow synthesis at near room temperature condition, which further improves the cost‐effectiveness.

### 3.1 Solid‐State Reaction (SSR)

**Figure 2:**
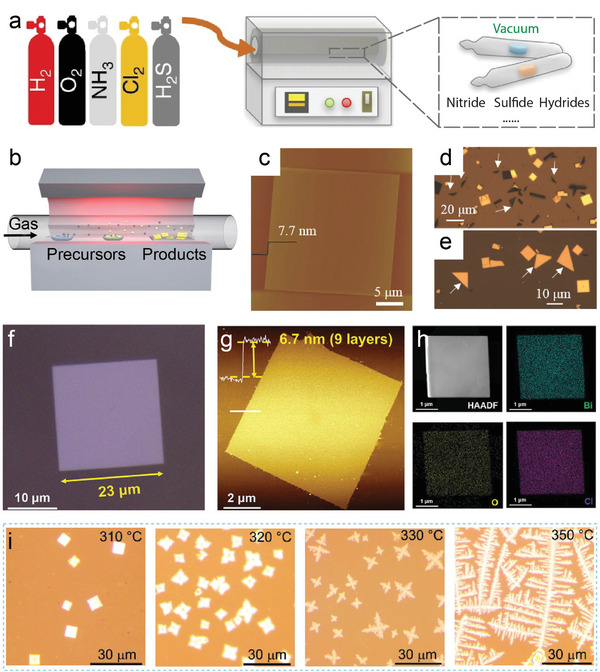
a) Schematic illustration of solid‐state reaction. Reproduced with permission.^[^ ^21^ ^]^ Copyright 2018, Springer Nature. b) Schematic illustration of CVD process. Reproduced with permission.^[^ ^39^ ^]^ Copyright 2020, American Chemical Society. c) A representative AFM image of a single Bi_2_O_2_Se nanoplate with a thickness of 7.7 nm. Optical image (OM) of (d) triangular Bi_2_O_x_Se sheets vertically grown on mica substrate, and (e) triangular Bi_2_O_x_Se sheets mechanically pushed over, indicated by white arrows. Reproduced with permission.^[^ ^59^ ^]^ Copyright 2018, Wiley‐VCH. f) OM and (g) AFM image of a BiOCl flake. h) HAADF image and EELS elemental maps for Bi, O, and Cl. Reproduced with permission.^[^ ^21^ ^]^ Copyright 2020, Wiley‐VCH. i) OMs of BiOBr flakes at different growth temperatures (310, 320, 330, 350 °C), Ar 20 sccm, O_2_ 5 sccm, 10 min, 30 Torr. Reproduced with permission.^[^ ^5^ ^]^ Copyright 2020, American Chemical Society.

Solid‐State Reaction (SSR) is a conventional method for synthesizing layered anion‐mixed compounds. This method involves mixing and grinding solid precursors, such as metals and their compounds in a stoichiometric ratio. The resulting mixture is then pressed into pellets, which are sealed in an evacuated quartz tube and heated at high temperatures for a few days. **Figure** **2**a shows the schematics of SSR process. The SSR method offers high yield and solvent‐free conditions.

Drasar et al. reported the synthesis of Bi_2_O_2_Se using the SSR method, which involves calcination at temperatures of 1000–1050 K for 1–2 months.^[^ ^49^ ^]^ This process has been optimized using the spark plasma sintering (SPS) method, which applies a pulsed electric field to the powder mixture under pressure, resulting in rapid heating and sintering of the material. Consequently, the same synthesis can now be achieved at 898 K in several hours.

As the complexity of the compounds increases, obtaining high phase‐purity anion‐mixed oxycompounds becomes more challenging. For quaternary compounds, the potential challenge for multi‐phase formation due to inhomogeneous chemical compositions is significant. Li et al. addressed this issue by synthesizing single‐phased BiCuSeO using SSR integrated with a ball milling process which efficiently improved compositional homogeneity and promoting complete reactions compared to hand grinding.^[^ ^28^ ^]^

When synthesizing more complex quinary oxycompounds, the process typically involves using metal sulfides and metal oxides as precursors for solid‐state reactions to provide suitable reactivity to form a desired complex structure. For example, Cao and colleagues utilized La_2_S_3_, Bi_2_S_3_, and PbO as starting materials to synthesize LaPbBiS_3_O. The complete reaction involved two heating stages, 940 K for 1500 min, followed by 1020 K for 1500 min.^[^ ^33^ ^]^ This two‐stage heating process allows for the initial formation and homogenization of intermediate phases at a lower temperature, followed by further crystallization and purification at a higher temperature, ensuring the growth of high‐quality crystals.

Moreover, the traditional SSR method can be modified by incorporating flux‐assisted calcination (FAC), where a fluxing agent is added to the mixture of solid reactants. The fluxing agent facilitates the reaction by lowering the melting points of the reactants or intermediates and enhancing diffusion rates.

For ternary anion mixed oxycompounds, Zheng's group reported the successful synthesis of single crystals of DyOCl with dimensions up to 3 × 2 × 0.05 mm^3^ using this approach.^[^ ^50^ ^]^ In their method, a mixture of Dy_2_O_3_ and DyCl_3_ in a mole ratio of 1:15 was prepared, with DyCl_3_ serving as the flux. The mixture was heated to 1100 °C and maintained at this temperature for 24 h. During this high‐temperature reaction, the DyCl_3_ flux lowered the melting point of the mixture, enabling the formation of a liquid‐phased chloride at a lower temperature. Subsequently, the mixture was cooled to 700 °C at a rate of 2 °C h^−1^. This controlled cooling process facilitated the crystallization of DyOCl and led to the growth of transparent single crystals. The excess flux was removed by dissolving it in water.

For the quaternary example, Chen's group successfully demonstrated the feasibility of BiCuSeO crystal using the flux‐assisted method.^[^ ^51^ ^]^ The synthesized Bi_2_O_2_Se is mixed with Cu_2_Se powder in a 1:1 molar ratio. This mixture is combined with an equal molar proportion of NaCl and KCl to serve as the flux. To prevent the decomposition of BiCuSeO at high temperatures, all materials are placed in an alumina crucible inside a fused silica tube. The tube is evacuated to ≈4 × 10^−6^ Torr and sealed to create an anaerobic environment. The starting material and flux are heated to the selected temperatures of 775, 730, and 690 °C and kept for 24 h to ensure complete reaction. The melted reactance is then slowly cooled to 600 °C over 15 days, allowing for the controlled crystallization of BiCuSeO. The final cooling to room temperature is achieved naturally. Once the sample is cooled, the melt is dissolved in distilled water, separating the shiny, thin, plate‐like BiCuSeO crystals from the solvent.

For more complex quinary example, Cao's group reported the utilization of flux‐assisted method to produce the high‐quality Ba_2_Ti_2_Fe_2_As_4_O crystal.^[^ ^38^ ^]^ In their study, the process begins with the preparation of the Ba_2_As_3_ flux, which is prepared by reacting Ba and As in a sealed quartz tube. This tube is heated to 700 °C at a rate of 30 °C h^−1^ and held for 10 h. The resulting flux is then mixed with other starting materials, specifically TiO_2_, Ti, and Fe, in a molar ratio of TiO_2_:Ti:Fe:Ba_2_As_3_ = 1:3:4:6. This mixture is pressed into a pellet and placed in an alumina crucible, which is subsequently sealed in an evacuated quartz tube. All operations are performed in a glove box to maintain extremely low water and oxygen levels to prevent contamination. The mixture is heated to 1150 °C and held for 10 h, allowing the reactants to fully melt and react in the presence of flux. Following this, the mixture is slowly cooled to 900 °C at a rate of 2.5 °C h^−1^. The as‐prepared crystals are then washed in alcohol to remove the Ba_2_As_3_ flux, leaving behind shiny plate‐like crystals. To enhance superconductivity, these crystals are further annealed at 500 °C in a vacuum for a week. The flux‐assisted method plays a crucial role in lowering the melting point, enhancing diffusion rates, and enabling controlled crystallization, resulting in the formation of high‐quality crystals with improved properties.

In summary, while the SSR method offers the advantage of high yield through facile synthesis in a solvent‐free condition, it has limitations in achieving homogeneity in the chemical composition of the final product, which can lead to the formation of multiple phases and affect material properties. Additionally, SSR requires high reaction temperatures and long reaction times due to the reactivity constraints of the starting materials, limiting its efficiency. The incorporation of flux‐assisted methods addresses these issues by promoting the diffusion of ions and atoms, significantly enhancing the growth of larger, well‐formed single crystals compared to what would typically be achieved through SSR alone.

### 3.2 Polymerized Complex Reaction (PC) Enabled Method

The Polymerized complex (PC) method is a two‐step synthesis technique particularly effective for synthesizing layered quaternary oxycompounds. First, polymerized complex reaction is used to prepare one of the reactants. The resulting polymer complex is then calcined for obtaining the final product. The PC method is advantageous due to its ability to produce smaller product sizes, and its lower energy consumption compared to other synthesis techniques.

Domen's group first developed the PC‐based two‐step route to prepare Sm_2_Ti_2_S_2_O_5_ with a short heating time of 1 h at a low temperature of 473 K. The energy consumption is greatly reduced compared to single‐step SSR synthesis.^[^ ^52^ ^]^ The final product has size range of 0.5‐5.0 µm, with primary particles having diameters of 0.1–0.5 µm and a specific surface area of 6 m^2^ g^−1^. In comparison, the SSR‐synthesized counterparts had a size range of 2–4 µm and a lower specific surface area of 0.6 m^2^ g^−1^. The smaller size and larger surface area could lead to less recombination of electron‐hole pairs which have the ability to enhance photocatalytic activity. Abe et al. later successfully synthesized sillen‐aurivillius oxychloride Bi_4_TaO_8_Cl using the PC method, incorporating a calcination step with BiOCl.^[^ ^53^ ^]^ In this study, methanol and citric acid were used as the organic monomers to conduct the polymerization. The resulting particles with a small size of 100–300 nm had a high specific surface area of 4.0 m^2^ g^−1^ compared to the conventional ones with a size of over 500 nm using SSR.

The PC method offers advantages in terms of lower energy consumption, and control over particle size and morphology, making it particularly suitable for synthesizing complex compounds. However, the purity of the final product can be compromised using organic precursors, which may lead to residual organic contamination.

### 3.3 Chemical Vapor‐Based Method

#### 3.3.1 Chemical Vapor Deposition (CVD)

Chemical Vapor Deposition (CVD) is a popular method for preparing mono‐ or few‐layered materials on substrates. In a typical process, precursors and substrates are placed separately in a furnace tube. Carrier gases transport precursor vapors under controlled temperature and pressure which causes the precursor vapors to react or decompose, eventually depositing the material onto the substrate, as shown in Figure 2b. The CVD method offers a high‐purity product and precise control over its thickness. However, given the complexity of the deposition process, the CVD method is more widely explored in synthesizing ternary layered material.

For the oxychalcogenides, A noteworthy study by Peng and co‐workers demonstrated the successful preparation of 2D Bi_2_O_2_Se crystal using Bi_2_O_3_ powder as the bismuth source, placed at the hot center of the furnace, while Bi_2_Se_3_ bulk acted as the selenium source, positioned 5 cm upstream. The growth time ranged from 10 to 60 min. Despite the high time consumption, Peng's group has demonstrated a final product of lateral size over 200 µm which is the highest among all the CVD Bi_2_O_2_Se.^[^ ^54^ ^]^ Later, He et al. further investigated the growth of Bi_2_O_2_Se and vertical triangular Bi_2_O_x_Se nanoflakes using a low‐pressure CVD (LPCVD) method under different substrate temperatures.^[^ ^55^ ^]^ They demonstrated that substrate temperature influences the growth direction of Bi_2_O_2_Se, resulting in nanosheets with an ideal stoichiometric ratio and lateral arrangement. At higher substrate temperatures, Bi_2_O_2_Se exhibited lateral arranged rectangular nanosheet (Figure 2c–e). This phenomenon is attributed to the increased migration rate of adatoms at elevated temperatures, which favors the formation of thermodynamically stable structures. For 2D layered materials, the most stable structures are those terminated with (00n) planes due to the least number of dangling bonds in the direction. Conversely, at lower substrate temperatures, a kinetics‐dominated synthesis environment is established, in which the nanoribbon structure Bi_2_O_x_Se grows vertically along the (110) plane with reduced substrate restrictions and energy required (Figure 2c–e).

Moving from oxychalcogenides to oxyhalides, Yang and colleagues first reported the growth of vertically aligned hexagonal FeOCl nanosheets on fluorine‐doped tin oxide (FTO) glass substrates using CVD.^[^ ^56^ ^]^ In their procedure, anhydrous FeCl_3_ was used as the precursor and placed in an evaporator equipped with a spiral stainless steel tube. The evaporator was heated to 300 °C to evaporate the FeCl_3_ to form vertically aligned hexagonal FeOCl nanosheet on the FTO substrate. Later, Zhai et al. reported the synthesis of the largest 2D BiOCl layers using a salt‐assisted CVD method. BiOCl mixed with a small amount of NaCl was used as the precursor.^[^ ^57^ ^]^ The addition of salts to the precursors increases the reaction rate and lowers the reaction temperature. The precursor mixture was placed in the central heating zone of a furnace, which was heated to 900 °C with carrier gas of argon. The resulting BiOCl flakes deposited on a mica substrate exhibited a regular square 2D flake morphology, with the largest size reported to date of over 23 µm and a thickness of 6.7 nm, which is equivalent to ≈9 layers of the BiOCl (Figure 2f,g). Electron energy‐loss spectroscopy (EELS) is used to study the chemical composition of BiOCl flakes. The scanning maps for Bi, O, and Cl in Figure 2h reveal that the element distribution is uniform without large‐area lattice defects.

Soon after this, Xiao's group developed a one‐step CVD technique to grow 2D BiOBr nanosheets on various substrates including quartz, SiO_2_/Si, and FTO.^[^ ^58^ ^]^ The one‐step CVD method employed BiBr_3_ powder as the precursor placed in the upstream heating zone of a two‐zone furnace. High‐purity argon at 20 sccm and oxygen at 5 sccm were used as carrier and reaction gases, respectively. The source was heated to 310–350 °C and substate was at 200 °C. The growth process lasted 10 min at a pressure range of 10–50 Torr. It was found that longer time and higher pressure will lead to increased coverage and branch length. In particular, the source temperature also affects the material morphology (Figure 2i). At 310 °C, the BiOBr flakes exhibited a regular square shape with lateral sizes ranging from 5 to 20 µm. While at 320 °C, it produced dart‐shaped BiOBr flakes. At temperatures above 330 °C, the flakes developed dendritic structures with four prominent axes, consistent with the symmetry of the BiOBr crystal. The branch length of the dendritic structures could reach up to 200 µm, significantly larger than the compact square flakes.

In the study by Sun et al., the synthesis of bismuth oxyhalide thin films was achieved using mist CVD method at ambient pressure. The process involved nebulizing solutions containing BiCl_3_, BiBr_3_, or BiI_3_ to form microscale mists. The growth temperatures were optimized at 350 °C for BiOCl and 300 °C for BiOBr and BiOI. The carrier gas flow rate was set at 20 sccm of argon and 5 sccm of oxygen. SrTiO_3_ (STO) and (LaAlO_3_)_0.3_–(SrAl_0.5_Ta_0.5_O_3_)_0.7_ (LSAT) (001) single crystal substrates were used. This method can produce atomically flat, highly crystalline oxyhalides thin films.

In conclusion, the CVD method offers significant advantages for synthesizing high‐purity, well‐controlled thickness mono‐ and few‐layered materials. It is particularly beneficial for advanced electronic applications due to its precise control over film thickness and morphology. However, the complexity and cost associated with the deposition process, along with slower deposition rates, can limit its scalability for industrial applications. The growth and product details of representative studies are summerized in **Table** **2**.

**Table 2:** Summary of growth conditions of CVD for synthesis 2D layered metal oxychalcogenides and oxyhalides.

Precursors	Carrier gases	Substrate	Growth condition	Products	Lateral size	Thickness	Ref.
FeCl_3_	N_2_/O_2_	FTO	270°C, 30 min	FeOCl	1 µm	50‐60 nm	[56]
VCl_3_	Ar/H_2_	SiO_2_/Si	600°C, 10 min	VOCl	5 µm × 15 µm	10 nm	[60]
BiBr_3_	Ar/O_2_	quartz, SiO_2_/Si, or FTO	10‐50 Torr, 200°C, 10 min	BiOBr	5–20 µm	3.2–36.2 nm	[5, 58]
BiOCl	Ar	Mica	Ambient pressure, 450°C, 10 min	BiOCl	Over 23 µm	0.74–6.7 nm	[21, 57]
Bi_2_O_3_, Bi_2_Se_3_	Ar	Mica	100‐400 torr, 500–550°C, 10–60 min	Bi_2_O_2_Se	Over 200 µm	0.8–6.7 nm	[54]
Bi_2_O_3_, Bi_2_Se_3_	Ar	Mica	650‐700°C, 10–30 min	Bi_2_O_2_Se	7–11 µm	Less 10 nm	[55, 59]
Bi_2_O_3_, Bi_2_Se_3_	N_2_	Mica	2000 Pa, 520–600 °C	Bi_2_O_2_Se	Over 30 µm	7.2–10.2 nm	[39, 61]

#### 3.3.2 Chemical Vapor Transportation (CVT)

The Chemical Vapor Transport (CVT) method involves converting a solid material into its vapor phase and subsequently redepositing it in a different location via a chemical reaction facilitated by a transport agent. This technique enables the precise control of material growth and is commonly used for the synthesis and purification of crystalline materials.^[^ ^62^ ^]^ Unlike CVD, the CVT method requires a sealed tube for cleaner reaction zone. The material is transported from a hot zone for vaporization to a cool zone for deposition. The process may be aided by transport agent if the energy required for transportation is higher than the thermal force created by the temperature difference. In general, the reaction time for CVT spans several days, resulting in the growth of highly pure bulk crystal products. Given its high purification capabilities, the bulk products grown by CVT can be subjected to mechanical exfoliation. The resulting mono‐ and few‐layered flakes are highly desirable for use in modern electronic and optoelectronic devices. Given the complexity of CVT, the current studies are more focused on ternary oxycompounds.

**Figure 3:**
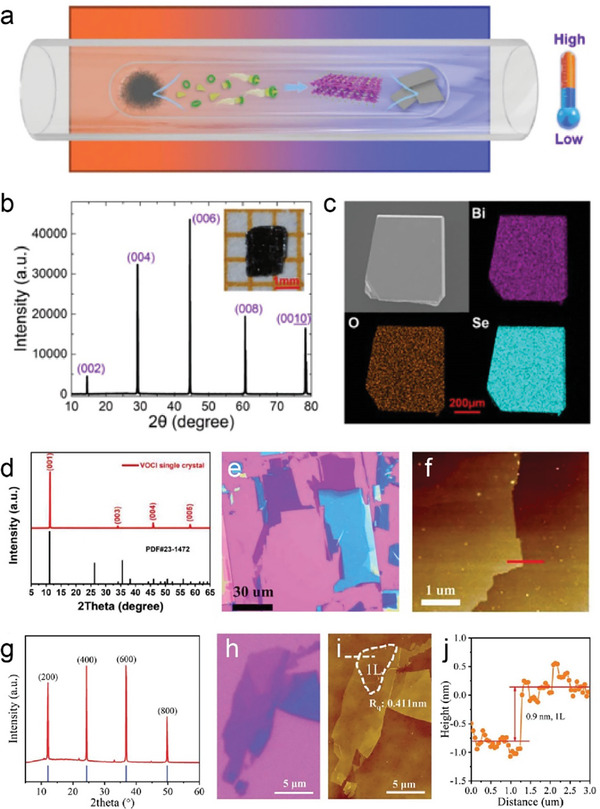
a) Schematic of the CVT growth equipment. b) XRD of the Bi_2_O_2_Se crystal. Inset: typical photograph of the as‐grown Bi_2_O_2_Se. c) SEM image and Bi (purple), O (orange), and Se (azure) elemental mapping of Bi_2_O_2_Se. Reproduced with permission.^[^ ^66^ ^]^ Copyright 2019, American Physical Society. d) XRD patterns of VOCl crystals. e) The optical image of VOCl flakes. f) AFM image of a thin VOCl flake. Inset: thickness profile of the VOCl flake corresponding to the red line. Reproduced with permission.^[^ ^24^ ^]^ Copyright 2020, IOP Publishing. g) Powder X‐ray pattern of the NbOI_2_ crystals. h) Optical image and (i) AFM images of as exfoliated 2D NbOI_2_ flake. j) The thickness of monolayer NbOI_2_. Reproduced with permission.^[^ ^27^ ^]^ Copyright 2021, Wiley‐VCH.

Chen and colleagues utilized the CVT method to synthesize highly crystalline Bi_2_O_2_Se nanoplates, as shown in the schematic **Figure** **3**a. Using a sealed quartz tube and a two‐zone furnace, they achieved millimeter‐sized crystals over 10 days.^[^ ^63^ ^]^ As shown in Figure 3b, the sharp peaks of the Bi_2_O_2_Se crystal in the X‐ray diffraction (XRD) pattern indicate the high crystalline quality of Bi_2_O_2_Se. The EDS mappings of Bi, Se, and O elements in the Bi_2_O_2_Se crystal (Figure 3c) present the homogeneous distribution of all the elements. Lin et al. followed a similar approach but avoided transport agents, enhancing purity and reducing unintentional doping.^[^ ^64^ ^]^ Similarly, Song et al. prepared bulk VOCl single crystals with a length of 0.5 cm. In their method, V_2_O_3_ and VCl_3_ were enclosed within a quartz tube filled with propane and oxygen. The sealed tube was subsequently placed in a two‐zone furnace with a temperature gradient ranging from 850 °C to 750 °C and maintained for 5 days.^[^ ^24^ ^]^ The single‐crystal XRD (Figure 3d) shows the diffraction patterns of the (00l) plane. Interestingly, the bulk VOCl crystals were able to be exfoliated into a square‐like few‐layer with a thickness of 3.7 nm by scotch tapes as shown in Figure 3e,f. In addition, high‐purity NbOI_2_ single crystals plates with dimensions of 6 × 8 × 0.5 mm^3^ were grown through the CVT method and mechanically exfoliated into thin flakes which were then transferred onto a SiO_2_/Si substrate using poly (dimethylsiloxane) (PDMS) film.^[^ ^27^ ^]^ The XRD pattern with four peaks including (200), (400), (600), and (800) in Figure 3g indicates the preferential growth orientation of NbOI_2_ is (100) plane. Monolayers with a thickness of 0.9 nm were successfully obtained and confirmed by atomic force microscopy (AFM), as shown in Figure 3h–j.

Though the CVT technique extends the reaction time, it achieves bulk crystal with higher purification and less defects. The crystal obtained can be readily exfoliated onto substrate thanks to their intrinsic vdW layered crystal nature.^[^ ^24^, ^27^, ^63^, ^64^, ^65^ ^]^ Given the significance of 2D‐centric research across a broad field of science, the CVT method emerges as a compelling strategy to create 2D oxycompound materials, particularly the ones with large scale production capability and high crystal quality.

### 3.4 Hydro/Solvothermal Method

**Figure 4:**
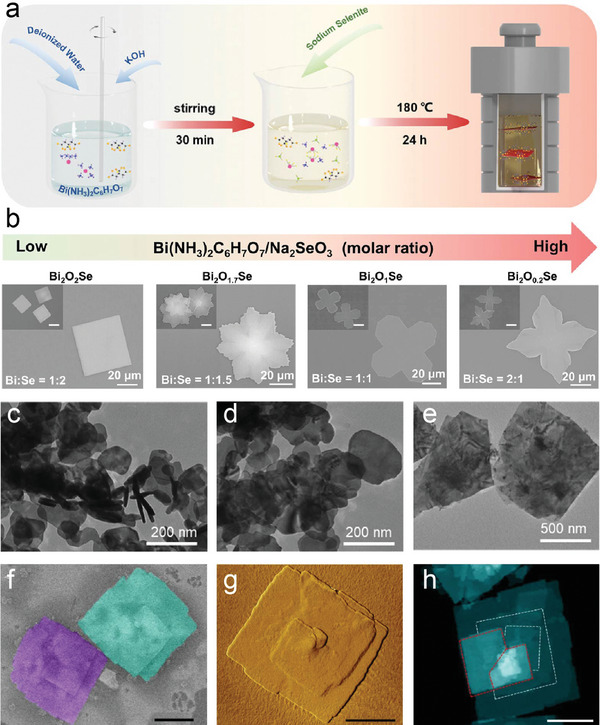
a) Schematic of hydro/solvothermal method. b) SEM images of typical Bi_2_O_x_Se nanosheets grown with molar ratios of Bi to Se = 1:2, 1:1.5, 1:1, and 2:1 (from left to right), respectively. Reproduced with permission. Copyright 2022, Wiley‐VCH.^[^ ^68^ ^]^ TEM images of the Bi_2_O_2_Se nanosheets obtained from different amounts of LiNO_3_ c) 0 g, d) 3 g, e) 10 g. Reproduced with permission.^[^ ^69^ ^]^ Copyright 2020, Royal Society of Chemistry. f) SEM, g) AFM, and h) HAADF image of BiOCl nanosheets with the spiral shape. Scale bar: 500 nm. Reproduced with permission.^[^ ^74^ ^]^ Copyright 2019, Springer Nature.

Hydrothermal and solvothermal methods are two methods used to prepare oxycompounds under controlled conditions, each utilizing distinct environments and solvents. Hydrothermal synthesis takes place in aqueous solutions under an elevated temperature and under high pressures. In contrast, solvothermal method occurs in organic solvents at high temperatures and pressures, allowing precise control over reaction parameters such as temperature and solvent composition. Water serves as both a reactant and solvent, facilitating controlled dissolution and precipitation processes. The standard procedure of the hydro/solvothermal method is illustrated in **Figure** **4**a. Solvothermal method allows for precise control over reaction conditions, whereas hydrothermal synthesis enhances crystallization and optimizes the properties of the final product.

Bismuth oxychalcogenides have been successfully synthesized using either a hydrothermal or solvothermal approach. For instance, Huang's group reported the synthesis of Bi_2_O_2_S. Bi(NO_3_)_3_·5H_2_O, CH_4_N_2_S, and LiOH·H_2_O were dissolved in deionized water.^[^ ^17^ ^]^ The solution was then sealed in a Teflon‐lined stainless‐steel autoclave and maintained at 200 °C for 3 days. This controlled environment allowed for the formation of Bi_2_O_2_S nanoplate with uniform size and good crystallinity. In another study, Liu and colleagues reported on a hydrothermal route for fabricating freestanding Bi_2_O_2_Ch nanosheets.^[^ ^67^ ^]^ Here, C_6_H_13_BiN_2_O_7_·H_2_O served as the source of Bi, while either thiourea or sodium selenite acted as sources of S or Se, respectively. The resulting Bi_2_O_2_S and Bi_2_O_2_Se nanosheets exhibited lateral sizes exceeding 2.0 µm and thicknesses of ≈5.0 nm. Furthermore, Li and co‐workers recently pioneered an innovative organic ion template‐guided solution growth for 2D Bi_2_O_2_Se, achieving the largest domain sizes exceeding 60 µm reported to date.^[^ ^68^ ^]^ They employed Bi(NH_3_)_2_C_6_H_7_O_7_ as the bismuth source, which dissociates in the solution to release bismuth ions along with organic citrate ions (C_6_H_5_O_7_)^3−^. These citrate ions act as templates by creating a confined environment where the nucleation and growth of Bi_2_O_2_Se nanosheets can occur. The organic ion templates selectively associated with metal ions, leading to the oriented growth of Bi_2_O_2_Se crystals with the support of surface poisoning. Furthermore, NH_3_ generated through hydrolysis plays a crucial role in the growth of 2D Bi_2_O_2_Se crystals as well. It not only moderates the rate of crystal growth due to the weak reduction capability but also serves to adjust the pH of the solution, leading to high‐quality crystal growth and formation of diverse morphologies. Besides, the morphologies and chemical compositions of as‐grown Bi_2_O_2_Se nanosheets exhibit a correlation with the molar ratios of precursors Bi(NH_3_)_2_C_2_H_5_OH and Na_2_SeO_3_, resulting in a gradual evolution from a square to a four‐pointed star morphology as the Bi(NH_3_)_2_C_2_H_5_OH to Na_2_SeO_3_ molar ratio increases (Figure 4b). The organic ion templates selectively associate with metal ions, facilitating the oriented growth of Bi_2_O_2_Se crystals with support from surface poisoning mechanisms.

In addition to the effects of organic ion templates and precursor ratios, the influence of other ionic species on the growth of Bi_2_O_2_Se nanosheets has also been explored. Qu et al. found that the presence of Li^+^ can effectively control the lateral size (from 0.15 to 1 µm) of Bi_2_O_2_Se nanosheets with a thickness of ≈5 nm during the hydrothermal process (Figure 4c–e).^[^ ^69^ ^]^ This is due to the preferential adsorption of Li^+^ on {001} facets, leading to an inhibited growth rate along the [001] direction.

The synthesis of bismuth oxyhalides has been validated by numerous studies. For example, Tang's group demonstrated the ability to control the morphology of BiOX (where X = Cl, Br) by employing hydrothermal methods. Their research showed that by varying reaction temperature and precursor concentrations, they are able to produce BiOX in different morphologies, including nanoplates, nanosheets, and microsheets. The introduction of surfactants, such as polyvinylpyrrolidone (PVP) and cetyltrimethylammonium bromide has been shown to enhance the formation of 2D layers of BiOX synthesis, due to the absorbance on specific crystal planes that promotes the growth of the layer.^[^ ^70^ ^]^

For instance, metal Bi particles were oxidized by hydrogen peroxide in a DI water solution containing PVP and sodium chloride or sodium bromide, followed by hydrothermal treatment. This process yielded BiOX nanosheets with in‐plane sizes ranging from 2 to 50 µm and thicknesses of 40 to 80 nm. In contrast, Zhang's study has made significant progress in synthesizing BiOX with ultrathin thicknesses of 2.4 nm (three layers) and domain sizes ranging from 50 to 200 nm, featuring selective facets. This was achieved using Bi(NO_3_)_3_·5H_2_O, saturated NaBr solution, PVP, and mannitol through a mild solvothermal process.^[^ ^4^ ^]^ Lu et al. further extended this route to prepare few‐layered BiOX (X = Cl, Br, I) nanosheets using NaX salts as precursors.^[^ ^71^ ^]^ They demonstrated a facile hydrothermal method to obtain BiOBr nanosheets with different morphologies in square and circular shapes by adjusting the pH of the reactive solution with NaOH.^[^ ^72^ ^]^ The BiOBr nanosheets prepared in this study exhibited two different morphologies, namely BiOBr‐square with inner strain and BiOBr‐circle with relatively strain‐free, and their lateral sizes are several micrometres while the thicknesses are 31 and 22 nm, respectively. A recent study reported a one‐pot solvothermal method for the bottom‐up synthesis of homogeneous BiOCl, a wide‐bandgap 2D semiconductor, with good reproducibility.^[^ ^73^ ^]^ Unlike previous methods which typically involve directly stacking two monolayers, this approach produced BiOCl by chemically growing spiral nanosheets driven by screw dislocations in a scalable and straightforward process. The resulting BiOCl nanosheets had a twist angle of 1.6‐3.0° between adjacent layers, with an average size of 1.2 µm, as shown in Figure 4f–h.

Although the hydrothermal and solvothermal methods have shown promise in the preparation of bismuth oxychalcogenides and oxyhalides, there are still limitations that exist. The products obtained may suffer from organic contamination, restricted crystal size, and poor crystallinity. Additionally, the scalability of these methods can be limited by the need for high‐pressure and high‐temperature conditions, which may not be feasible for large‐scale production.

### 3.5 Post‐Synthesis–Liquid Exfoliation

Liquid exfoliation involves the dispersing of bulk materials in a suitable solution, which is then subjected to sonication, along with various mechanical or chemical techniques, to separate the bulk materials into individual layers.^[^ ^75^ ^]^ This method can produce high‐quality 2D nanosheets in large quantities. In the case of anion‐mixed oxycompounds, liquid exfoliation has been typically adopted in ternary oxycompound as the procedure to break the synthesized parental bulk counterpart into 2D layers.

**Figure 5:**
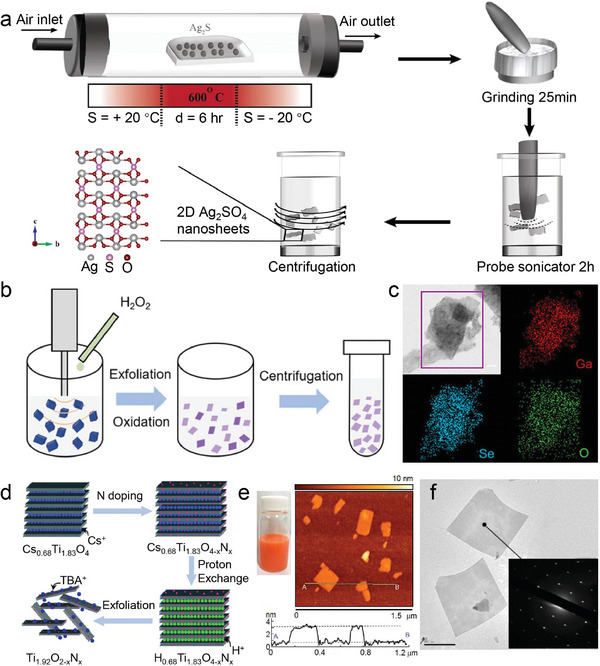
a) Schematic diagram for preparing 2D Ag_2_SO_4_ nanosheets. Reproduced with permission.^[^ ^78^ ^]^ Copyright 2022, Royal Society of Chemistry. b) Schematics of in situ mechanical preparation for 2D gallium oxyselenide. c) The TEM image of gallium oxyselenide and corresponding EDS mapping. Scale bar: 20 nm. Reproduced with permission.^[^ ^81^ ^]^ Copyright 2023, Elsevier. d) Schematic of procedures for preparing nitrogen‐doped Ti_0.91_O_2_ nanosheets. TBA^+^: tetrabutylammonium ion. Reproduced with permission.^[^ ^86^ ^]^ Copyright 2009, Royal Society of Chemistry. e) The appearance of a nanosheet suspension and AFM image of Ca_2_Ta_3_O_9.7_N_0.2_ nanosheets. f) TEM image and SAED pattern (inset) of Ca_2_Ta_3_O_9.7_N_0.2_ nanosheets. Reproduced with permission.^[^ ^87^ ^]^ Copyright 2012, American Chemical Society.

For instance, the oxychalcogenides Bi_2_O_2_Ch compounds have a unique characteristic where their atomic layers are held together by electrostatic interactions instead of weak vdW forces, making their exfoliation particularly challenging.^[^ ^16a^ ^]^ However, Wang's group innovatively prepared the Bi_2_O_2_Se nanosheets using a kitchen blender via a shear exfoliation.^[^ ^75^ ^]^ The Bi_2_O_2_Se powder was initially synthesized using SSR and then subjected to shear exfoliation. The resulting Bi_2_O_2_Se nanosheets had a lateral size of ≈500 nm and a thickness of 20–30 layers. In addition, both Bi_2_O_2‐2x_Te_2x_Se with Te substitution at the O site and Sb‐doping Bi_2_O_2_Se pellets were also prepared by shear exfoliation followed by SPS.^[^ ^76^ ^]^ Moreover, atomically‐thin transition metal sulfates [MSO_4_; M = Metal] including palladium sulfate (PdSO_4_),^[^ ^77^ ^]^ silver sulfate (Ag_2_SO_4_),^[^ ^78^ ^]^ nickel sulfate (NiSO_4_),^[^ ^79^ ^]^ and cobalt sulfate (CoSO_4_)^[^ ^80^ ^]^ have been investigated in recent year for the possibility of subject to liquid exfoliation. During these processes, the metal sulfide bulks are thinned down to ultrathin nanoflakes in the presence of strong mechanical forces and/or elevated annealing temperatures. In an oxygen‐enriched environment, the S in the original sulfide framework can be oxidized, forming sulfate ion (SO_4_)^2−^ to re‐bond with the metal atoms.^[^ ^77^, ^80^ ^]^ Building on a creative mechanism, Ou's group proposed a two‐step annealing‐exfoliation strategy that successfully facilitated the synthesis of 2D Ag_2_SO_4_. In this methodology, bulk Ag_2_S underwent annealing in air to transform into Ag_2_SO_4_. This is followed by exfoliation, driven by strong mechanical forces within the solvent phase to produce the desired 2D structure (**Figure** **5**a).^[^ ^78^ ^]^ To simplify the tedious steps, a single‐step based liquid exfoliation method was developed for preparing ultrathin gallium oxyselenides^[^ ^64^, ^81^ ^]^ and p‐type 1D/2D heterostructure consisting of Se belts and GeSe_x_O_y_^[^ ^82^ ^]^ nanosheets (Figure 5b). Interestingly, the level of Se atom substitution with O atoms in gallium oxyselenides nanosheets is modulated by the concentration of hydrogen peroxide. As shown in Figure 5c, the low‐magnification TEM image and corresponding EDS images depicted the high quality of gallium oxyselenides nanosheets.

In the scope of oxyhalides, FeOCl is a typical oxycompound investigated for its potential to be exfoliated using the liquid exfoliation method. Chen et al. confirmed the feasibility of exfoliation by calculating the cleavage energy and solvent surface tension using the first‐principles calculations. Then, they exfoliated the FeOCl plates prepared by SSR into few‐layer nanosheets using ultrasonication in acetonitrile solvent.^[^ ^83^ ^]^ The obtained nanosheets had a thickness of 1.40–3.37 nm corresponding to one to four layers. Besides, ions’ intercalation is a common strategy to assist liquid exfoliation. Interestingly, the polycrystalline bulk FeOCl with lithium intercalation produces Li_x_FeOCl can be exfoliated into few‐layered nanosheets in a methanol (MeOH) solvent.^[^ ^84^ ^]^ Similarly, lithium intercalation with shear force‐assisted liquid phase exfoliation was also applied to fabricate few‐layer Bi_2_O_2_Se nanoflakes with lateral dimensions of 20–60 nm and a thickness of 2.8 nm.^[^ ^85^ ^]^

In another study related to oxynitrides, Lu's group has reported a two‐step procedure to obtain nitrogen‐doped titania nanosheets with a thickness of ≈1 nm and a lateral size of sub‐micrometre. The layered titanate compounds were first doped with nitrogen by heating in an NH_3_ atmosphere and then exfoliated into single‐layer titanium oxynitride (N‐doped titania) nanosheets (Figure 5d).^[^ ^86^ ^]^ Following a similar procedure, bication oxynitride tantalum‐based oxynitride [Ca_2_Ta_3_O_9.7_N_0.2_]^−^ monolayer nanosheets with a thickness of 2.8‐3.1 nm and lateral dimensions of 100–500 nm were synthesized by Ida and co‐workers (Figure 5e and f). The large intercalated tetrabutylammonium ions act as the cations balancing the charge of [Ca_2_Ta_3_O_9.7_N_0.2_]^−^ nanosheets.^[^ ^87^ ^]^

In summary, while liquid exfoliation is capable of producing nanosheets on a large scale, the challenge of achieving uniform size and high homogeneity among the nanosheets remains. However, with further development and optimization of liquid exfoliation strategies, it is possible to obtain large‐scale, uniform, and high‐quality nanosheets of layered materials with multiple anions. One promising approach involves selecting solvents that are compatible with the surfaced energy of the bulk materials, as this can enhance the exfoliation efficiency and produce nanosheets with superior quality and sizes. Another strategy includes the use of aqueous solutions with polymers or surfactants to stabilize the exfoliated nanosheets, thereby preventing re‐aggregation. Additionally, modifying the mechanical parameters of the exfoliation process to ensure that large pieces of material are not fractured during the exfoliation. With these advanced strategies, liquid exfoliation has the potential to become a significant method for the synthesis of 2D materials, offering distinct properties for various applications in optoelectronics, catalysis, and energy conversion.

### 3.6 Other Methods

In addition to the methods mentioned above, several other techniques were also used to prepare few‐layer or monolayer single‐metal oxyhalides and oxychalcogenides. These methods include molecular beam epitaxy (MBE),^[^ ^88^ ^]^ pulse laser deposition (PLD),^[^ ^89^ ^]^ solution‐assisted method,^[^ ^90^ ^]^ and microwave‐assisted method.^[^ ^91^ ^]^

**Figure 6:**
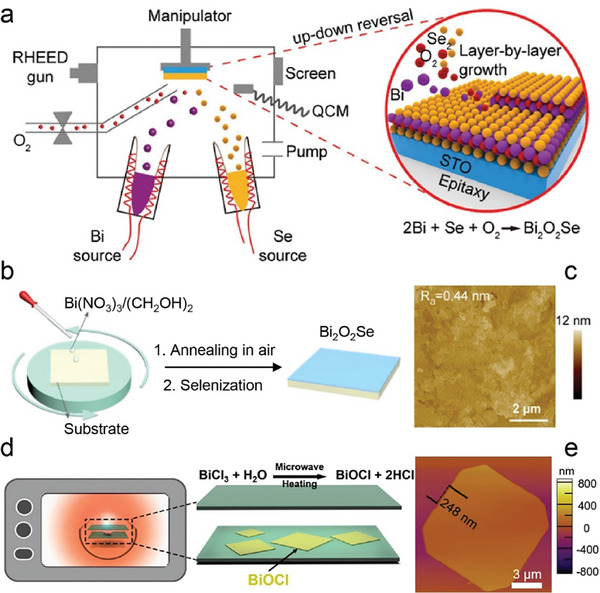
a) Schematic diagram of MBE growth of atomically thin Bi_2_O_2_Se films on a (001)‐oriented SrTiO_3_ (STO) substrate by MBE. Reproduced with permission.^[^ ^88^ ^]^ Copyright 2019, Wiley‐VCH. b) Solution preparation process of the Bi_2_O_2_Se thin film. c) Typical AFM image of a Bi_2_O_2_Se thin film with a relatively small roughness. Reproduced with permission.^[^ ^90^ ^]^ Copyright 2020, American Chemical Society. d) Schematic illustration of the space‐confined microwave synthesis of BiOCl crystals. e) AFM topography of BiOCl crystal and corresponding height profile. Reproduced under terms of the CC‐BY license.^[^ ^91^ ^]^ Copyright 2019, The Authors, published by Wiley‐VCH.

MBE is a remarkable technology that utilizes ultra‐high vacuum environments and ultra‐low growth rates to precisely control the thickness of products.^[^ ^92^ ^]^ With this method, Peng et al. obtained thin films of Bi_2_O_2_Se on SrTiO_3_ substrate by co‐evaporating Bi and Se precursors in an oxygen atmosphere at 290 °C (**Figure** **6**a).^[^ ^93^ ^]^

In preparation for epitaxial growth of Bi_2_O_2_Se films, STO (001) substrates are pretreated under ultra‐high vacuum at 950 °C, resulting in a TiO_2_‐terminated surface with well‐defined terraces and a step height of 0.4 nm. During the initial growth stage, monolayer Bi_2_O_2_Se islands nucleate along these terraces, forming discontinuous 2D islands. As growth continues, these islands merge to create a continuous monolayer film. A Bi_2_O_2_Se monolayer is defined as half a unit cell, with a thickness of 0.6 nm. With increased growth coverage, additional 2D islands emerge, maintaining regular orientations aligned with the STO substrate's lattice direction. The growth proceeds in a 2D mode until a complete unit cell‐thick Bi2O2Se film is achieved, after which further growth leads to thicker films (≈20 nm). Another method for producing thin films from bulk targets in a high vacuum is the PLD method. PLD has been used to successfully produce thin films of several oxychalcogenides, including Bi_2_O_2_Se and LnCuOCh (Ln = La‐Nd; Ch = S‐The).^[^ ^89^, ^94^ ^]^ Overall, the primary advantages of MBE and PLD lie in their ability to precisely control the thickness and composition of the deposited films, as well as their reproducibility and product scale.

The solution‐based method is a facile, rapid, and scalable way to synthesize oxycompounds with multiple anions. Peng et al. developed a method for direct synthesis of Bi_2_O_2_Se thin films on flexible substrates using a two‐step process. This involves the decomposition of Bi(NO_3_)_3_·5H_2_O and selenization of Bi_2_O_3_, as illustrated in Figure 6b.^[^ ^90^ ^]^ The resulting Bi_2_O_2_Se film exhibits a smooth surface and maintains excellent structural and electrical stability even under bending conditions (Figure 6c). This method is particularly suitable for the production of thin films with cost‐effectiveness, scalability, and compatibility with various substrates.

Additionally, Liu and co‐workers demonstrated a simple and reliable microwave‐assisted space‐confined approach to synthesizing 2D ternary‐layered BiOCl crystals in a short time of < 3 min (Figure 6d).^[^ ^91^ ^]^ Here, BiCl_3_ powder as the precursor was placed in a confined space created by two stacking SiO_2_/Si substrates which were then heated by microwave. The as‐synthesized BiOCl nanoflakes possess a lateral size of 10 µm and a thickness of 248 nm (Figure 6e). When the SiO_2_/Si substrate was replaced with mica, the thickness was reduced to 8.7 nm. The unique microwave heating facilitated quick and efficient reaction of the perishable precursors in unsuitable conditions, offering a novel strategy to prepare specific materials.

Currently, the solution‐mediated biphasic sequential approach and the microwave‐accelerated spatially restricted method have exclusively been employed in the synthesis of Bi_2_O_2_Se and BiOCl, respectively the potential to extend these methodologies to create a wide range of alternative 2D oxygenated compounds opens up enormous opportunities for exploration.

In order to provide a comprehensive overview, we have summarized the preparation methods of the reported layered anion‐mixed oxycompounds in **Table** **3**.

**Table 3:** Summary for the synthesis method of layered oxycompounds.

Method	Precursor	Products	Morphology	Ref.
SSR	Y_2_O_3_, Y_2_S_3_, TiO_2_	Y_2_Ti_2_O_5_S_2_	Particle Several micrometres ‐10 µm	[3a]
SSR	Bi_2_O_3_, Cu, Bi, and Se powders	BiCuSeO	Bulk	[28]
SSR	Bi_2_O_3_, Bi, Se	Bi_2_O_2_Se	Bulk	[95]
SSR	La_2_S_3_, Bi_2_S_3_ and PbO	LaPbBiS_3_O	Bulk	[33]
PC	Ti(OiPr)_4_ and Ln(NO_3_)_3_·6H_2_O Ethylene glycol, methanol, anhydrous citric acid	Ln_2_Ti_2_S_2_O_5_	Bulk	[96]
PC	Ti(OiPr)_4_ and Sm(NO_3_)_3_·6H_2_O Ethylene glycol, methanol, anhydrous citric acid	Sm_2_Ti_2_S_2_O_5_	Bulk	[97]
PC	Bi(NO_3_)_3_·5H_2_O, TaCl_5_, ethylene glycol, citric acid, BiOCl	Bi_4_TaO_8_Cl	Particle 100–300 nm	[53]
CVD	Bi_2_O_3_ powder, Se powder, Bi powder	Bi_2_O_2_Se	‐^a)^	[7, 98]
CVD	Bi_2_O_3_, Bi_2_Se_3_	Bi_2_O_2_Se	Nanoribbons Thickness down to 0.65 nm	[99]
LPCVD^b)^	Bi_2_O_3_, Bi_2_Se_3_	Bi_2_O_2_Se	Nanosheets Thickness below 10 nm; Lateral size 7–11 µm	[59]
LPCVD	Bi_2_O_3_, Bi_2_Se_3_	Self‐standing Bi_2_O_2_Se	Nanoplates Thickness 20–30 nm	[100]
CVD	FeCl_3_	FeOCl	Nanosheets Thickness 50–60 nm; Lateral size 1 µm	[56, 65]
CVD	BiOCl powder	BiOCl	Flakes Thickness 0.74–6.7 nm; Lateral size 23 µm	[21]
CVD	BiBr_3_ powder	BiOBr	Flakes Thickness 3.2–36.2 nm; Lateral size 5–20 µm	[5]
CVT	Bi_2_O_2_Se polycrystalline powders, I_2_	Bi_2_O_2_Se	Nanoplates	[65a]
CVT	V_2_O_5_, VCl_3_	VOCl	Bulk	[24]
CVT	Nb, Nb_2_O_5_ and I_2_ powders	NbOI_2_	Bulk	[27]
Liquid‐Exfoliation	FeOCl plates	FeOCl	Nanosheets Thickness 1.40–3.37 nm	[83]
Liquid‐Exfoliation	FeOCl powder	FeOCl	Nanosheets Thickness 2.0–2.4nm Lateral size 0.5–1.0 µm	[84]
Liquid‐Exfoliation	Bi_2_O_2_Se powders	Bi_2_O_2_Se	Nanosheets Thickness 20–30 layers Lateral size ≈500 nm	[75]
Liquid‐Exfoliation	Bi_2_O_2_Se powders	Bi_2_O_2_Se	Flakes Thickness 2.8 nm; Lateral size 20–60 nm	[85]
Liquid‐Exfoliation	Cs_0.68_Ti_1.83_O_4_	Ti_0.91_O_2−x_N_x_	Nanosheets Thickness ≈1 nm; Lateral size sub‐micrometer	[86]
Liquid‐Exfoliation	CsCa_2_Ta_3_O_9.7_N_0.2_	[Ca_2_Ta_3_O_9.7_N_0.2_]^−^	Nanosheet Thickness 2.8–3.1 nm; Lateral size 0.1–0.5 µm	[87]
Solvothermal Pressure‐Relief Process	La(NO_3_)_3_·nH_2_O, CH_4_N_2_S	La_2_O_2_S	Nanoparticles	[101]
Hydrothermal	Bi(NO)_3_·5H_2_O, CH_4_N_2_S, KOH, LiOH	Bi_9_O_7.5_S_6_/ Bi_2_O_2_S	Bulk	[17, 20]
Hydrothermal	Bi particles, PVP, NaCl, H_2_O_2_	BiOX (X = Cl, Br)	Nanoplates Thickness: 10–30 nm; Lateral size: 200–500 nm Nanosheets Thickness: 40–80 nm; Lateral size: 2–50 µm Microsheets Thickness: 200–1000 nm; Lateral size: 10–100 µm	[70]
Hydrothermal	Bi(NO_3_)_3_·5H_2_O, KCl, NaOH	BiOCl	Nanosheets Thickness: 80–200 nm; Lateral size: 1–3 µm,	[102]
Solvothermal	Bi(NO_3_)_3_·5H_2_O, NaBr, mannitol, PVP	BiOBr	Nanosheets Thickness: 2.4 nm; Lateral size: 50–200 nm	[4]
Hydrothermal	Bi(NO_3_)_3_·5H_2_O, mannitol, PVP, NaX (X = Cl, Br, I)	BiOX (X = Cl, Br, I)	Nanosheets	[71]
Hydrothermal	Bi(NO_3_)_3_·5H_2_O, cetyltrimethylammonium bromide, NaOH	BiOBr	Nanosheets Thickness: ≈30 nm; Lateral size: several micrometers	[103]
Solvothermal	Bi(NO_3_)_3_·5H_2_O, Poly(diallyldimethylammonium chloride), tetracycline	BiOCl	2D spiral nanosheets	[74]
Hydrothermal	Bi(NO_3_)_3_·5H_2_O, KNO_3_, LiNO_3_, Se	Bi_2_O_2_Se	Nanosheets Thickness ≈120 nm	[104]
Hydrothermal	C_6_H_13_BiN_2_O_7_·H_2_O, CH_4_N_2_S, Na_2_O_3_Se	Bi_2_O_2_S/Bi_2_OSe	Nanosheets Thickness: ≈5.0 nm; Lateral size: over 2.0 µm	[67]
Hydrothermal	Bi(NO_3_)_3_, Na_2_Se, LiNO_3_	Bi_2_O_2_Se	Nanosheets Thickness: 5 nm; Lateral size: 0.5–1 µm	[69]
MBE	Bi, Se, O_2_	Bi_2_O_2_Se	Atomically thin film	[88]
PLD	Bi_2_O_2_Se targets	Bi_2_O_2_Se	Film Thickness: 23 nm	[89]
Solution‐assisted method	Bi(NO_3_)_3_·5H_2_O, selenium pills	Bi_2_O_2_Se	Thin film	[90]
Mist CVD	BiCl_3_ or BiBr_3_ or BiI_3_	BiOCl, BiOBr, BiOI	Thin film	[105]
Space‐confined microwave	BiCl_3_, H_2_O	BiOCl	Platelets Thickness: 248 nm, Lateral size: 10 µm	[91]

^a)^ Not mentioned;

^b)^ Low pressure chemical vapor deposition.

### 3.7 Discussion and Conclusion

First, the SSR method offers high yield and is solvent‐free but faces challenges such as low reactant efficiency and potential multiphase formation, which can compromise the purity of materials. The incorporation of flux‐assisted calcination can enhance ion diffusion, resulting in larger, well‐formed single crystals.^[^ ^50^ ^]^

In comparison, CVD and its variants, including LPCVD and Mist CVD, provide high purity and precise control over film thickness and morphology. They are particularly suitable for producing high‐quality films with excellent material properties, essential for advanced electronic applications. Despite these advantages, these methods tend to be complex and costly, with slower deposition rates that may limit scalability.

The CVT method is distinguished by its ability to grow large, high‐purity crystals, which, despite requiring extended reaction times, are ideal for bulk crystal production and subsequent exfoliation into few‐layer materials.

When considering techniques that provide unparalleled control over film thickness and composition, MBE and PLD stand out. MBE, in particular, is known for producing high‐quality, atomically precise films under ultra‐high vacuum conditions. Both methods, however, are expensive and have slow deposition rates, which can constrain their use in large‐scale manufacturing.

Hydrothermal and solvothermal methods are valued for their simplicity, low cost, and fast processing times, making them attractive for large‐scale production. These methods excel in controlling particle size and morphology, although they may suffer from organic contamination and limited crystal size.

However, the solution‐assisted methods and microwave‐assisted methods provide facile, fast, and scalable approaches to synthesizing mixed anion oxycompounds. Solution‐assisted methods are cost‐effective and compatible with various substrates, utilizing standard glassware and operating at lower temperatures and pressures. Typically, hydrothermal methods require autoclaves and operate under higher temperatures and pressures. Microwave‐assisted methods offer rapid synthesis with excellent control over material properties. These methods are particularly advantageous for flexible and scalable production.

In conclusion, each synthesis method for mixed anion oxycompounds has its own set of benefits and disadvantages, with the choice of method depending on the specific requirements of the application. By leveraging the strengths of each method and addressing their limitations, it is possible to optimize the synthesis for a wide range of scientific and industrial applications.

## 4 Properties and Applications

### 4.1 Electronic, Optical Properties and Applications

#### 4.1.1 Electronic and Optical Properties

**Figure 7:**
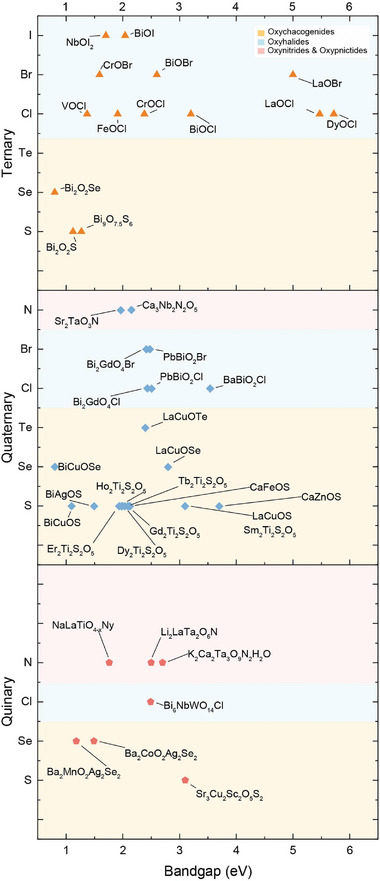
The bandgaps of various layered mixed‐anion oxycompounds.

Layered anion‐mixed oxycompounds cover semiconductors, insulators, and superconductors. Most oxychalcogenides, oxyhalides, and oxynitrides are semiconductors with a narrower bandgap compared to their oxide counterparts. This narrowing of the bandgap is attributed to the hybridization of oxygen *p* orbitals with those less electronegative anions, which results in the negative shift of the valence band.^[^ ^3a^ ^]^ Such bandgap narrowing enhances their photocatalytic performance under visible light which is an important component of solar energy. In addition, ternary lanthanum‐based oxyhalides such as LaOCl and LaOBr are insulators with wide bandgap exceeding 5 eV. Combining with their high dielectric constants allows them excellent candidates for gate dielectrics. Furthermore, there are some superconductors including oxychalcogenides with [BiS_2_] blocks and iron‐based oxypnictides with [FePn] blocks. The bandgaps of layered anion‐mixed oxycompounds are summarized in **Figure** **7**.

**Figure 8:**
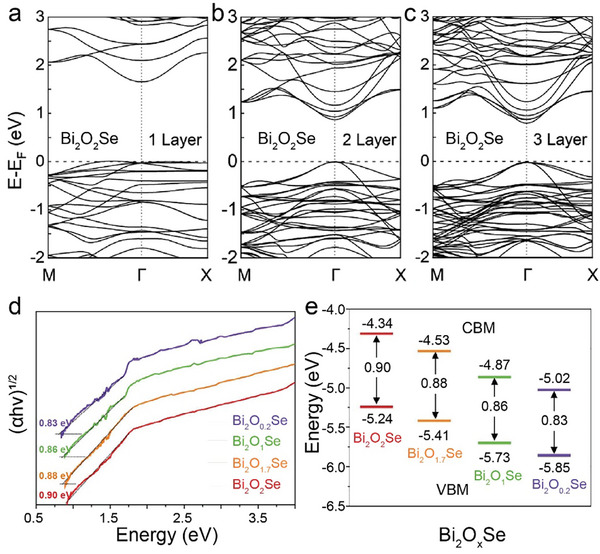
Band structures of Bi_2_O_2_Se with different layers. a–c) Band structure of Bi_2_O_2_Se with layer numbers from 1 to 3. Reproduced with permission.^[^ ^1a^ ^]^ Copyright 2019, American Chemical Society. d) Bi_2_O_x_Se (x = 2, 1.7, 1, and 0.2) (αhν)^1/2^‐hν curves. e) Energy‐band alignments of different Bi_2_O_x_Se materials. Reproduced with permission.^[^ ^68^ ^]^ Copyright 2022, Wiley‐VCH.

Ternary metal oxycompounds mainly include Bi_2_O_2_Ch, BiOX, MOX_2_, and FeOX. The band structure characteristics and optical properties of the Bi_2_O_2_Ch system were analyzed by means of first‐principle calculations.^[^ ^106^ ^]^ Ma and colleague concluded that both the 6*p*‐orbital of the bismuth atoms and the 2*p*‐orbital of the oxygen atoms contribute to the conduction band minimum (CBM), while both the *p*‐orbital of chalcogen atoms and the 2*p*‐orbital of oxygen atoms contribute to the valence band maximum (VBM). However, the former contribution is consistently more significant in both the CBM and VBM. As the chalcogen atomic number becomes larger, such role of oxygen atoms is weakened. Hence, the 6*p*‐orbital of the bismuth atoms almost entirely affects the CBM of Bi_2_O_2_Te, while the 5*p*‐orbital of the tellurium atoms almost exclusively contributes to the VBM. The bandgap gradually narrows with the chalcogen atomic number geting larger, as seen in the case of Bi_2_O_2_S (1.27 eV), Bi_2_O_2_Se (0.85 eV), and Bi_2_O_2_Te (0.11 eV). This can be attributed to the anion in this series (S → Se → Te) having a lower *p*‐orbital ionization energy. Besides, the electronic band structure of Bi_2_O_2_Ch can be tunable by controlling layer thickness and oxygen defects which can result in quantum confinement effects.^[^ ^7^, ^68^, ^107^ ^]^ The band structure of Bi_2_O_2_Se has been evaluated by Peng and coworkers using first‐principles calculations and angle‐resolved photoemission spectroscopy (ARPES). The bandgap of Bi_2_O_2_Se gradually decreases with the increase in the thickness from monolayer to three layers, as shown in **Figure** **8**a–c. This thickness‐dependent phenomenon is similar to other classic 2D materials such as MoS_2_^[^ ^108^ ^]^ and black phosphorus.^[^ ^109^ ^]^ The behavior can be derived from the strong dispersion along the Γ‐X and Γ‐M directions of the electronic states near the CBM at the Γ point.^[^ ^7^ ^]^ The ARPES measurement revealed an indirect bandgap of 0.8 eV and a very low in‐plane electron effective mass of m*  =  0.14 ± 0.02*m*_0_ (*m*_0_ is the free‐electron mass), even lower than that of silicon (0.26*m*_0_).^[^ ^110^ ^]^ This implies the possibility of ultrahigh electron mobility. Moreover, the experiment also showed that electronic structures of Bi_2_O_x_Se (Bi_2_O_2_Se, Bi_2_O_1.7_Se, Bi_2_O_1_Se, and Bi_2_O_0.2_Se) could be tuned by the oxygen deficiency concentration.^[^ ^68^ ^]^ As shown in Figure 8d,e, when the oxygen deficiency concentration increases, the bandgap of Bi_2_O_x_Se decreases, while the work function increases. This is possible because of the empty filled states by electron transfer to the defect state oxygen.

In addition to tunable electronic band structures, Liang et al. have calculated the linear optical properties of Bi_2_O_2_Ch using a PBEsol method.^[^ ^106b^ ^]^ The calculated absorption spectra revealed that the optical properties of Bi_2_O_2_Ch are anisotropic, stemming from their structural anisotropic. This anisotropy in optical behavior, coupled with tunable electronic structures, renders 2D layered Bi_2_O_2_Ch highly appealing for future applications in electronic and optoelectronic devices.

Ternary oxychalcogenides, Bi_4_O_4_S_3_, exhibit metallic behavior. Its Fermi level is located at the peak position of the partial density of states of the Bi 6*p* orbitals of BiS_2_ layer. On the BiS_2_ layer, the band structure near the Fermi level is primarily composed of contributions from the *p_x_* and *p_y_* orbitals of Bi atom, which exhibit a quasi‐1D character. This quasi‐one‐dimensionality leads to good nesting at the Fermi surface, and this electronic characteristic may interact with bosonic modes that mediate pairing, potentially enhancing the pairing interaction. Interestingly, near the *R* point, these orbitals tend to hybridize and form a 2D band structure.^[^ ^40a^ ^]^ Bi_4_O_4_S_3_ is also a typical type II‐superconductor with electron‐type carriers,^[^ ^111^ ^]^ exhibiting superconductivity with an onset transition temperature ($T_{c}^{onset}$) of 4.5 K and a zero resistivity temperature (*T*_*c*0_) of 4.02 K at zero magnetic field (*H* = 0 T).

**Figure 9:**
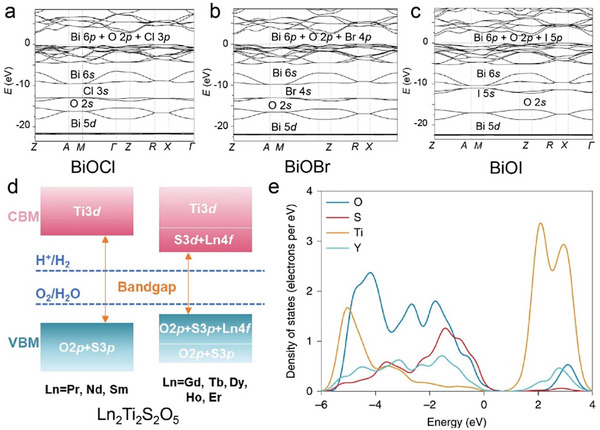
The band structures of a) BiOCl, b) BiOBr, and c) BiOI crystals. Reproduced with permission.^[^ ^129^ ^]^ Copyright 2008, Wiley‐VCH. Schematic band structure of d) Ln_2_Ti_2_S_2_O_5_ Reproduced with permission.^[^ ^96^ ^]^ Copyright 2004, American Chemical Society. e) DOS for O (blue line), S (red line), Ti (orange line), and Y (light blue line) of Y_2_Ti_2_S_2_O_5_. Reproduced with permission.^[^ ^3a^ ^]^ Copyright 2019, Springer Nature.

Similar to bismuth oxychalcogenides, the bandgap of bismuth oxyhalides, BiOX (X = Cl, Br, I), decreases with the increasing of atomic numbers: BiOCl (2.50 eV), BiOBr (2.10 eV), and BiOI (1.59 eV), as illustrated in **Figure** **9**a–c.^[^ ^112^ ^]^ The higher electronegativity of halogen atoms, compared to chalcogen atoms, results in their *p*‐orbitals being located at lower energies. This characteristic imparts wider bandgaps in halogen‐containing BiOX materials than in their chalcogen counterparts.^[^ ^113^ ^]^ Zhao et al. explored the relationship between intrinsic structural characteristics and optical properties of BiOX via density functional theory (DFT).^[^ ^114^ ^]^ They found that with the contribution of X n*s* states in BiOX's densities of states increasing, the efficiency of photogenerated electron transitions gradually improves, and benefits for charge generation and transfer. Meanwhile, the absorption edges for BiOCl, BiOBr, and BiOI crystals were calculated to be 355, 448, and 645 nm, respectively. Zhang et al. further performed the UV–vis experiment to estimate the adsorption edges of BiOX powders and obtained similar results to the calculated values (370, 440, and 670 nm).^[^ ^115^ ^]^ These phenomena demonstrate the potential of BiOX for photocatalytic applications. BiOX is composed of [Bi_2_O_2_]^2+^ slabs sandwiched between two sheets of X^2−^ with an internal electric field. This unique layered structure of BiOX allows the efficient separation of photogenerated holes and electrons into distinct atom planes. This internal electric field of BiOX in a unique layered structure not only brings high performance in photocatalytic applications but also enables the non‐linear optical (NLO) property.^[^ ^116^ ^]^ As reported by Li and coworkers, BiOCl nanosheets with preferentially exposed {001} facets (BiOCl {001}) and {010} facets (BiOCl {010}) were investigated using Z‐scan measurements.^[^ ^116b^ ^]^ And it was found that BiOCl {010} had an effective nonlinear absorption coefficient, which was ≈1.5 times higher than that of BiOCl {001}. Charge localization in BiOCl {010} was identified as the reason for its superior performance compared to BiOCl {001}, highlighting the significant role that excited‐state absorption plays in the reverse saturable absorption behavior of BiOCl nanosheets.

LnOX (Ln = La, Dy; X = Cl, Br, I) are insulators with wide bandgaps exceeding 5 eV. Regarding the wide bandgap, LaOCl and LaOBr are not only predicted to the promising high‐*κ* dielectrics using first principle methods but also utilized for gate dielectrics to realize high‐performance FET devices.^[^ ^117^ ^]^

In a study by Wang et al., the FeOX (X = F, Cl, Br, I) monolayers were calculated to be indirect Mott insulator semiconductors with bandgaps from 2.73 eV to 0.48 eV by GGA+U (U_eff_ = 5.3 eV) method.^[^ ^118^ ^]^ The bandgaps of the FeOF, FeOCl, and FeOBr monolayers, ranging from 1.5 to 3.0 eV, exceed the threshold of 1.23 eV, making them suitable for photoelectronic applications within the visible spectrum.^[^ ^119^ ^]^ Moreover, the band dispersions of VBM along the S‐Y and the S‐X directions of the Brillouin zone for FeOF and FeOCl, and the dispersions of VBM along the Γ‐X and the Γ‐Y directions for FeOBr and FeOI are different. These signs implicitly reflect the anisotropy of the effective mass of holes in 2D FeOX.

Layered oxide dichlorides MOX_2_ (M = V, Nb, Mo, Ru, and Os; X = Cl, Br, I) possess various electronic densities *n* for the M atoms (*n* = 1, M = V and Nb; *n* = 2, M = Mo; *n* = 4, M = Ru and Os).^[^ ^26^, ^120^ ^]^ The crystal structure of MOX_2_ consists of MO_2_X_4_ octahedra with the 1D M‐X chain geometric structures along the *b‐axis* and the M‐O geometric chain along the *a‐axis*, enabling their anisotropic behavior.^[^ ^41c^ ^]^ The anisotropic bonds along in‐plane contributes to a Peierls‐distorted polar structure that is associated with optical properties.^[^ ^27^ ^]^ Most notably, NbOI_2_ flakes with 20 nm thickness demonstrated an absolute SHG conversion efficiency of 0.006%,^[^ ^8^ ^]^ which is orders of magnitude higher than values reported for other 2D materials,^[^ ^9a,b^ ^]^ Weyl semi‐metals,^[^ ^121^ ^]^ and conventional nonlinear materials.^[^ ^9e^ ^]^ With an average pump intensity of 8 kW cm^−2^, an absolute SHG conversion efficiency of >0.2%, and an effective nonlinear susceptibility $\chi_{eff}^{\left(2\right)}$ in the order of 10^−9^ m V^−1^ were reported.^[^ ^8^ ^]^ Furthermore, the nonlinear response scales of MOX_2_ can be tuned with layer thickness, strain, and electricity.

Layered quaternary oxychalcogenides are usually semiconductors.^[^ ^122^ ^]^ For example, Ln_2_Ti_2_S_2_O_5_ and LaCuOCh are typical layered quaternary oxycompounds with bandgaps of ≈2 eV. Conductive bands of earlier lanthanide‐based oxysulfides [Pr, Nd, Sm]_2_Ti_2_S_2_O5 consist of Ti 3*d* without mixing with O 2*p* and S 3*p* orbitals. While lanthanoid elements’ 4*f* orbitals of Ln_2_Ti_2_S_2_O_5_ (Gd, Tb, Dy, Ho, Er) hybridize with O 2*p* and S 3*p* orbitals in the valence band and hybridize with S 3*p* orbitals in the conduction band as illustrated in Figure 9d.^[^ ^96^ ^]^ This is due to the lowering energy of the 4*f* orbital that promotes the hybridization of orbitals. As a result, the bandgap energy of Ln_2_Ti_2_S_2_O_5_ decreases from 2.13 eV to 1.94 eV as the atomic number of lanthanide ions increases from 62 (Sm^3+^) to 68 (Er^3+^). Such favorable bandgaps of 2 eV make them the potential candidate for visible‐light‐driven photocatalytic applications. The partial density of states of Y_2_Ti_2_S_2_O_5_, an analog of Ln_2_Ti_2_S_2_O_5_, were computed by the Cambridge Serial Total Energy Package (Figure 9e).^[^ ^3a^ ^]^ The CBM of Y_2_Ti_2_O_5_S_2_ is mainly composed of Ti 3*d* orbitals, while the VBM is formed by a combination of O 2*p*, S 3*p*, and Y 3*d* orbitals. Notably, there is a considerable overlap between the S 3*p* band and states originating from O 2*p* orbitals, indicating the hybridization between S and O atoms. Another material, LaCuOCh has a band structure similar to the lanthanide‐based oxysulfide. However, compared to other LnCuOS compounds, CeCuOS is easily oxidized, becoming copper‐deficient and exhibiting a narrow bandgap (<1eV). The susceptibility of CeCuOS to oxidation led to inaccuracies in early studies of its lattice parameters and electronic structure, as these investigations were conducted on non‐stoichiometric CeCuOS.^[^ ^42b^ ^]^ This oxidation process could result in the oxidation of Ce^3+^ or the antibonding states at the upper end of the Cu 3*d*/S 3*p* band, which easily forms a depleted valence band in LaCuOS. Chan et al.’s calculation indicates that the Ce 4*f* states are situated near the Fermi level above the Cu 3*d*/S 3*p* state, suggesting that copper deficiency can lead to mixed‐valent state of Ce.^[^ ^42b^ ^]^ It is worth noting that LaCuOCh is not an efficient photocatalyst due to its high effective mass (>1.0 *m_e_*), which contributes small hole mobility (≪10 cm^2^ V^−1^ s^−1^). To overcome this obstacle, non‐lanthanide Bi^3+^ ions were considered as a substitute for the lanthanum ions (Ln^3+^) due to the similar ionic radiuses and sharply different electronic configurations (Bi^3+^, [Xe] 4*f* ^14^5*d* ^10^6*s* ^2^6*p* ^0^; La^3+^, [Xe]4*f* ^0^5*d* ^0^6*s* ^0^).^[^ ^42a^ ^]^ The bandgap of BiCuOCh ranges from 0.84 to 1.1 eV, much narrower than that of LaCuOCh (2.4‐3.1 eV). This discrepancy arises from the fact that the conduction band in BiCuOCh is primarily composed of Bi 6*p* orbitals, whereas in LaCuOCh, it is determined by Cu 4*s* orbitals. Nonetheless, the VBM of both BiCuOCh and LaCuOCh are comprised of Cu 3*d* and Ch 2*p*.

Due to the coexistence of two distinct anions (O^2−^ vs. S^2−^, Se^2−^, or Te^2−^) in oxychalcogenides crystal structures, anion‐mixed functional groups have a much higher probability of accomplishing excellent NLO performances.^[^ ^123^ ^]^ Several layered oxychalcogenides have already been demonstrated to exhibit impressive SHG responses as Infrared (IR) NLO materials, such as CaZnSO,^[^ ^124^ ^]^ SrZn_2_OS_2_,^[^ ^125^ ^]^ Sr_8_Ga_8_O_3_S_14_,^[^ ^126^ ^]^ Sr_6_Cd_2_Sb_6_O_7_S_10_,^[^ ^127^ ^]^ and Sr_4_Pb_1.5_Sb_5_O_5_Se_8_.^[^ ^128^ ^]^

Quaternary oxyhalides are usually semiconductors. Bi_4_MO_8_X (M = Nb, Ta; X = Cl, Br) exhibits a unique band structure compared with ternary oxyhalides, BiOX (X = Cl, Br, I). DFT results indicate that the VBM is predominantly formed by oxygen 2*p* orbitals, rather than the n*p* orbitals of the more electronegative halogens.^[^ ^3^, ^130^ ^]^ This special structure can be explained by a Madelung site potential analysis.^[^ ^3c^ ^]^ The VBM of Bi_4_MO_8_X (Bi_4_NbO_8_Cl) originates from the oxygen in the fluorite‐based [Bi_2_O_2_] layer. Additionally, the presence of [NbO_4_] slabs leads to a higher density of oxygen around the [Bi_2_O_2_] layer, which contributes to the increased energy of the oxygen orbitals in Bi_4_NbO_8_X.

Oxynitrides represent a significant class of mixed‐anion compounds, characterized by the lower electronegativity of nitrogen compared to oxygen. This disparity results in a negative shift of the valence band positions due to the nitrogen *p* orbitals, thereby reducing the bandgap. Consequently, oxynitrides emerge as promising materials for visible light photocatalysis. Layered structures are rarely found in ternary oxynitrides but are more commonly observed in quaternary and quinary oxynitride compounds. For example, Sr_2_TaO_3_N is a representative layered quaternary oxynitride with a Ruddlesden‐Popper structure and a moderate bandgap value of 1.97 eV, which enables visible light absorption. Theoretical calculations indicate that Sr_2_TaO_3_N exhibits typical 2D charge transport properties due to its structural laminations. The confinement of electrons and holes within the TaN_2_O_2_ square planes enhances charge transport by minimizing charge recombination across different TaN_2_O_2_ square planes.^[^ ^30^ ^]^ Similarly, layered quinary oxynitrides are capable of absorbing visible light due to the hybridization of nitrogen and oxygen anions *p* orbitals, as seen in quaternary oxycompounds.

Among the layered quaternary oxypnictides, LaMOPn (M = Mn, Fe, Co, Ni; Pn = P, As) and Sr_2_Cr_3_As_2_O_2_ exhibit metallic behavior at ambient environment and superconductivity at certain low temperatures.^[^ ^131^ ^]^ The electronic transport property of LaMOP at 2 K varies significantly depending on the choice of transition metal M. When M = Mn (3*d* ^5^), it exhibits semiconducting behavior; when M = Fe (3*d* ^6^) or Ni (3*d* ^8^), it exhibits superconductivity; and when M = Co (3*d* ^7^), it behaves as a metal. LaFeOP differs from conventional layered copper‐based superconductors. In LaFeOP, the Fe^2+^ ions are located in a tetrahedral site, coordinated with four P^3−^ ions, forming the [FeP] layer through edge‐sharing tetrahedrons. In contrast, Cu^2+^ in the copper‐based superconductors is located in a planar 4‐fold square site. Therefore, electrons at the Fermi level occupy Cu^2+^ $3d_{x^{2}-y^{2}}\;$ orbitals for copper‐based superconductors. While the Fermi level is formed by the Fe^2+^ $3d_{z^{2}}\;$ and 3*d_xz_*/3*d_yz_* orbitals for LaFeOP.^[^ ^132^ ^]^ Particularly, Hideo Hosono's group achieved a high *T*_c_ of 26 K in LaFeAsO doped with fluorine (F), stimulating research interest into new iron‐based layered superconductors.^[^ ^31^, ^133^ ^]^

The quaternary oxypnictide Sr_2_Cr_3_As_2_O_2_, which has a Sr_2_Mn_3_As_2_O_2_‐type structure featuring both CrO_2_ planes and Cr_2_As_2_ layers, displays metallic property with an electron‐magnon scattering. First‐principles calculations reveal that the electronic states near the Fermi energy in this material are predominantly derived from Cr 3*d* orbitals, which exhibit weak hybridization with O 2*p* orbitals in the CrO_2_ layers and moderate hybridization with As 4*p* orbitals in the Cr_2_As_2_ layers. The calculated bare band structure density of states at the Fermi level is ≈1‐quarter of the experimental value obtained from low‐temperature specific heat measurements. The result is consistent with the significant electron‐magnon coupling observed. This characteristic, along with the structural properties, suggests the potential to induce superconductivity in Sr_2_Cr_3_As_2_O_2_ through chemical doping or high pressures.^[^ ^32^ ^]^

Quinary anion‐mixed oxycompounds have few types. Here we briefly introduce their electronic and optical properties. Quinary oxychalcogenides, LaPbBiS_3_O is a narrow gap semiconductor with an activation energy of ≈17 meV.^[^ ^33^ ^]^ Ba_2_Ti_2_Fe_2_As_4_O is a superconductor containing [Fe_2_As_2_] and [Ti_2_O] layers. First‐principles studies indicate that the intercalated [Ti_2_O] layers, with a possible charge or spin‐density wave ordering, transfer 0.12*e* of charge to the [Fe_2_As_2_] layers. This charge transfer induces superconductivity in the [Fe_2_As_2_] layers even in the absence of explicit doping.^[^ ^38^, ^134^ ^]^ Moreover, the annealing process leads to redistribution of Ti within the Fe planes, thereby enhancing superconductivity in the [Fe_2_As_2_] layers.^[^ ^38^ ^]^

#### 4.1.2 Applications

##### FET Channel Materials

In the last decade, there has been extensive research on Field‐Effect Transistors (FETs) based on 2D materials, including graphene, TMDs, and black phosphorus.^[^ ^135^ ^]^ More recently, the successful fabrication of monolayer Bi_2_O_2_Se with an ultrahigh Hall mobility of 28,900 cm^2^ V^−1^ s^−1^ at 1.9 K has garnered increased interest in layered oxychalcogenide materials.^[^ ^7^, ^136^ ^]^ The FET channel materials are mainly semiconductors.

**Figure 10:**
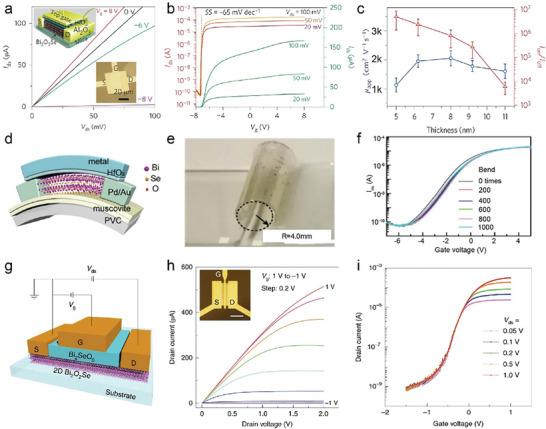
a) Output curves obtained from a 6.2 nm‐thick Bi_2_O_2_Se device at room temperature. Inset: schematic of the device structure (upper plane) and optical microscopy image of a top‐gated device (bottom plane) directly fabricated on a thick mica substrate. A 5 nm Al_2_O_3_ and 20 nm HfO_2_ serve as the single top‐gate dielectrics. b) Top‐gate transfer curves (I_ds_ ‐ V_g_) of the device with different source‐drain voltages (100, 50, 20 mV). I_ds_ are shown on a logarithmic scale (left) and a non‐logarithmic scale (right). c) *µ*_app_ and I_on_/I_off_ of Bi_2_O_2_Se FETs as a function of channel thickness. Reproduced with permission.^[^ ^7^ ^]^ Copyright 2017, Springer Nature. d) Device configuration of a flexible Bi_2_O_2_Se transistor on an ultrathin muscovite substrate with excellent flexibility. e) Photograph of flexible Bi_2_O_2_Se transistors on a muscovite/PVC substrate with a curvature radius (R) of 4.0 mm. f) Transfer characteristics of flexible Bi_2_O_2_Se transistors after 0, 200, 400, …, 1000 consecutive bending cycles at a 4.0 mm bending radius. Reproduced with permission.^[^ ^90^ ^]^ Copyright 2020, American Chemical Society. g) A 3D illustration of a Bi_2_O_2_Se FET with Bi_2_SeO_5_ as the gate dielectric. h) Output characteristics of a Bi_2_O_2_Se/Bi_2_SeO_5_ FET. Inset: optical microscopy image of the device. Scale bar, 20 µm. i) I_ds_‐V_g_ curves (transfer curves) of the FET. Reproduced with permission.^[^ ^141^ ^]^ Copyright 2020, Springer Nature.

Peng and co‐workers first developed FETs with excellent air stability and ultra‐high Hall mobility based on ultrathin films of non‐encapsulated layered Bi_2_O_2_Se (**Figure** **10**a–c). They found that Bi_2_O_2_Se crystal‐based top‐gated FETs exhibit a Hall mobility up to 450 cm^2^ V^−1^ s^−1^, a superior current on/off ratio of 10^6^, and a near‐ideal subthreshold swing value of ≈65 mV dec^−1^ at room temperature.^[^ ^7^ ^]^ Besides, the room temperature Hall mobility changes with different thicknesses, implying severer surface/interface electron scattering in a few layers Bi_2_O_2_Se flakes. Consequently, further efforts are required to enhance the performance of Bi_2_O_2_Se‐based FETs. A primary challenge is the presence of an unsatisfactory Schottky barrier between Bi_2_O_2_Se and metal electrodes, which results in suboptimal performance of Bi_2_O_2_Se FETs. Lu et al. explored metal‐bilayer Bi_2_O_2_Se interfacial properties by first‐principles band structure calculations and quantum transport simulation.^[^ ^137^ ^]^ They found that Pt, Sc, and Ti electrodes with bilayer Bi_2_O_2_Se showed a desirable lateral n‐type ohmic contact, which is contributed by the band hybridization of unusual Fermi‐level pinning above the monolayer Bi_2_O_2_Se conduction band. Furthermore, Yang et al. conducted theoretical evaluations of the upper‐performance limits of bilayer Bi_2_O_2_Se MOSFETs in ultrashort channels using *ab* initio quantum transport simulations.^[^ ^138^ ^]^ Their predictions indicate that with the use of underlap, bilayer Bi_2_O_2_Se n‐MOSFETs can meet the high‐performance criteria set by the ITRS 2028, even as the gate length is scaled down to 5 nm. This indicates a viable approach for developing sub‐10 nm FETs. In addition to suppressing short‐channel effects in nanoscale transistors, the exceptional flexibility of ultrathin 2D materials has made them a promising material for flexible electronics.^[^ ^139^ ^]^ Leveraging this characteristic, Peng et al. have developed a flexible top‐gated Bi_2_O_2_Se transistor on muscovite/PVC substrates. The device showcases remarkable electrical stability, maintaining a field‐effect mobility at 100 cm^2^ V^−1^ s^−1^ bent under different curvature radii and even after 1000 continuous bending cycles at a radius of 4.0 mm (Figure 10d–f).^[^ ^90^ ^]^ These findings highlight the potential of Bi_2_O_2_Se to be a strong candidate for future flexible electronic devices.

##### FET High‐κ Gate Dielectrics

As is known, the thin insulating layer of SiO_2_ can no longer meet the requirements of effective insulation along with the size shrinking of silicon‐based transistors. This leads to high leakage currents in transistors because electrons pass through SiO_2_ layers of less than 2 nm thick.^[^ ^140^ ^]^ Therefore, high‐κ gate dielectrics are in significant demand to maintain low leakage current and high gate capacitance. Most high‐κ gate dielectrics used in FETs are primarily insulating materials. It is worth noting that an ultra‐thin gate dielectric composed of Bi_2_SeO_5_ was obtained via layer‐by‐layer oxidization of the underlying high‐mobility 2D semiconducting Bi_2_O_2_Se. This process enables the creation of an atomically sharp and chemically clean interface when utilized in the fabrication of FET devices.^[^ ^141^ ^]^ The native oxide gate dielectric (Bi_2_SeO_5_) possesses a high dielectric constant of 21. As shown in Figure 10g‐i, devices based on this high‐κ gate dielectrics layer exhibited high apparent field‐effect mobilities of >300 cm^2^ V^−1^ s^−1^, high current on/off ratios of >10^5^, and low subthreshold swing values of ≈75 mV dec^−1^ at room temperature. Furthermore, Bi_2_SeO_5_ can also be selectively etched away by wet chemical methods, leaving the underlying Bi_2_O_2_Se almost unchanged. In addition, layered transition‐metal oxyhalide VOCl was also found with a high dielectric constant of 11.7.^[^ ^24^ ^]^ A back‐gated FET device was constructed based on MoSe_2_/VOCl/graphite heterostructure, which exhibited a current on/off ratio of 10^5^, electron mobility of 6.24 cm^2^ s^−1^ V^−1^, hole mobility of 0.18 cm^2^ s^−1^ V^−1^, a low subthreshold swing of 169 mV dec^−1^ and a low threshold voltage of 0.2 V at room temperature.

##### Photodetectors

Among the layered mixed‐anion oxycompounds, bismuth oxyhalides and oxychalcogenides possess an optimal bandgap for efficient absorption of solar radiation across a broad range of wavelengths of ultraviolet, visible, and near‐infrared (NIR) light. The layered mixed‐anion oxycompounds for photodetection are semiconductors. Due to this unique property, these materials have demonstrated immense potential in the development of optoelectronic devices that rely on the photoelectric effect.^[^ ^67^, ^142^ ^]^

**Figure 11:**
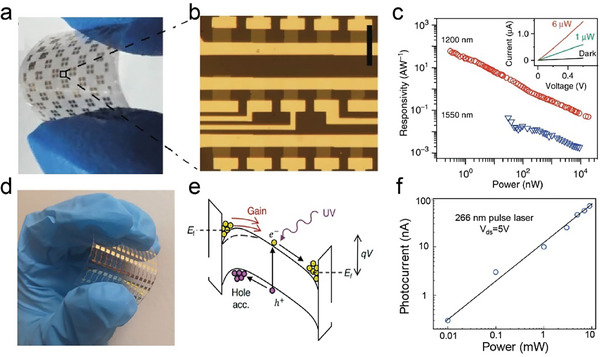
a) Illustration of photodetector based on 2D Bi_2_O_2_Se crystal, with the schematic crystal structure of alternatively stacked (Bi_2_O_2_)_n_ and Se_n_ layers. b) Left: photograph of 2D Bi_2_O_2_Se photodetectors and arrays on mica. Right: optical image of 3 × 5 multi‐pixel array of 2D Bi_2_O_2_Se photodetectors. Scale bar: 10 µm. c) Photoresponsivity under 0.6 V bias at wavelengths of 1200 nm and 1550 nm. Reproduced with permission.^[^ ^98^ ^]^ Copyright 2018, Springer Nature. d) The energy band diagram of the BiOCl detector under UV light illumination. e) The light response curve of the device under 266 nm laser illumination. f) Photocurrent measured at different incident laser pulse influences. Reproduced with permission.^[^ ^91^ ^]^ Copyright 2019, Wiley‐VCH.

2D bismuth oxychalcogenides possess environmental stability and a relatively small bandgap, making them suitable for use as visible and NIR photodetectors. Huang et al. fabricated visible‐light photodetectors based on bulk Bi_2_O_2_S with a bandgap of 1.12 eV.^[^ ^17^ ^]^ The device exhibited steady switching and reversible photo response at an “on” state of 373 µA cm^−2^ and an “off” state of 253 µA cm^−2^ at room temperature. Later, ultrathin Bi_2_O_2_S nanosheets synthesized by a one‐pot wet‐chemical method were also applied for NIR photodetection.^[^ ^143^ ^]^ The solution‐processed Bi_2_O_2_S nanosheets with a thickness of 2–3 nm have a bandgap of 1.5 eV, equal to 785 nm wavelength. The photodetector based on these ultrathin nanosheets showed a higher responsivity (4 A W^−1^) compared with that of bulk (0.373× 10^−3^ A W^−1^). It is worth noting that the device exhibited excellent air stability even after exposure to air for one month. Compared with Bi_2_O_2_S, layered 2D Bi_2_O_2_Se has a smaller bandgap of 0.8 eV, making it more suitable for photodetection in NIR and IR light regions. He and co‐workers reported the NIR photodetection properties of Bi_2_O_2_Se nanosheets synthesized using a low‐pressure CVD method.^[^ ^59^ ^]^ The photodetector exhibited high photoelectric performance with a response time of 2.8 ms, a responsivity of 6.5 A W^−1^, and a detectivity of 8.3 × 10^11^ Jones. Furthermore, the photodetector is stable even under a wide range of temperature changes (80–300 K). Later, Peng's group studied CVD‐prepared 2D Bi_2_O_2_Se IR photodetectors at room temperature.^[^ ^98^ ^]^ The 2D Bi_2_O_2_Se detector showed an extremely high sensitivity of 65 A W^−1^ at 1200 nm and had an ultra‐fast light response of ≈1 ps due to the high carrier mobility and suitable electronic bandgap. To verify the practical application, they fabricated flexible photodetectors on mica, which worked well under bending and remained robust when exposed to ambient air for up to 5 weeks (**Figure** **11**a–c). Recently, Wang et al. synthesized horizontally self‐standing Bi_2_O_2_Se nanostructures and assessed the performance of photodetectors based on self‐standing Bi_2_O_2_Se (ss‐Bi_2_O_2_Se) in comparison to those based on flat Bi_2_O_2_Se (f‐Bi_2_O_2_Se).^[^ ^100^ ^]^ The ss‐Bi_2_O_2_Se exhibited improved photoelectric performance with photoresponsivity and detectivity of 1.17 × 10^5^ A W^−1^ and 1.95 × 10^12^ Jones, respectively (2.50 × 10^4^ A W^−1^ and 2.84 × 10^11^ Jones for f‐Bi_2_O_2_Se), which could be attributed to the decreased defect‐related trap states.

2D layered BiOCl possesses a relatively large optical bandgap (3.2 eV), making it a suitable candidate for UV detectors. Liu et al. developed BiOCl‐based photodetectors operated at 266 nm laser.^[^ ^91^ ^]^ The device was demonstrated to be highly stable in the air, have a high responsivity of ≈8 A W^−1^ and ultrafast response time of ≈18 ps. They further transferred the BiOCl nanosheets on the PDMS substrate to fabricate flexible detector arrays (Figure 11d‐f), which shows potential for developing wearable UV devices. In a separate study, Zhai et al. also fabricated high‐responsivity solar‐blind ultraviolet (SBUV, 200–280 nm) photodetectors based on BiOCl flakes.^[^ ^21^ ^]^ The responsivity and detectivity of the BiOCl photodetectors could reach up to 35.7 A W^−1^ and 2.2 × 10^10^ Jones, respectively. Interestingly, increasing temperature can further improve the photo‐detective performance, with an elevated responsivity of 2,060 A W^−1^ at 450 K, which is attributed to the formation of defective BiOCl grains. In addition, Daeneke's group reported a UV detector based on 2D atomically thin and large‐area In_2_O_3−x_S_x_ nanosheets prepared by a wet chemical method.^[^ ^144^ ^]^ This resulting product showed a high photoresponsivity of ≈3400 A W^−1^ and a photodetectivity of ≈2.18 × 10^13^ Jones.

In summary, bismuth oxysulfides, oxyselenides, and oxychlorides‐based photodetectors have been studied extensively. However, these devices usually suffer from lower responsivity and slower response time, particularly in the case of Bi_2_O_2_S, which limits their potential in practical use. Thus, enhancing their performance through the exploration of heterostructures with different materials and examining other layered metal oxycompounds for their potential applicability in optoelectronic devices is crucial. **Table** **4** summarizes the key advancements made in the development of photodetectors that utilize layered bismuth oxyhalides and oxychalcogenides.

**Table 4:** Summary for magnetic properties of typical layered oxycompounds.

Materials	Structural type	Magnetic order	Magnetic order temperature	Ref.
FeOX (X = F, Cl, Br, I) monolayer	*Pmmn*	Antiferromagnetism	200 K	[118]
FeOCl nanosheets	*Pmmn*	Antiferromagnetism	14 K	[84]
FeOCl nanosheets	*Pmmn*	Antiferromagnetism	83.3 K	[1d]
VOCl flakes	*Pmmn*	Ferromagnetism	150 K	[189]
α‐VOCl_2_ monolayer	*Pmm2*	Antiferromagnetism	177 K	[41b]
CrOCl monolayer	*Pmmn*	Ferromagnetism	160 K	[186]
CrOBr monolayer	*Pmmn*	Ferromagnetism	129 K	[186]
CrOCl flakes	*Pmmm*	Antiferromagnetism	13.6 K	[10]
La_2_O_3_Mn_2_Se_2_	‐ ^a)^	Antiferromagnetism /Ferromagnetism	162 K	[188b]
Na_2_Fe_2_Se_2_O	–	Antiferromagnetism	73 K	[42d]
Sr_2_MnO_2_Cu_2‑δ_Se_2_	*I*4/*mmm*	Antiferromagnetism	54 K	[48d]
Sr_2_MnO_2_Ag_2‑δ_Se_2_	*I*4/*mmm*	Antiferromagnetism	67 K	[48d]
Sr_4_Mn_3_O_7.5_Cu_2_Ch_2_ (Ch = S, Se)	*I*4/*mmm*	Antiferromagnetism	110 K	[48j]
Ca_4_Fe_2_Cu_2_S_2_O_6_	*P*4/*nmm*	Antiferromagnetism	–	[48i]
Ba_2_CoO_2_Ag_2_Te_2_	*I*4/*mmm*	Antiferromagnetism	60 K	[48e]

^a)^ Not mentioned

##### Solar cells

Solar cells are optoelectronic devices that convert sunlight to electricity. Layered mixed‐anion oxycompound semiconductors are mainly used as absorber layers in solar cells. Bismuth oxychalcogenides such as Bi_2_O_2_S and Bi_2_O_2_Se possess appropriate bandgap between 0.8 and 1.9 eV, high carrier mobility, and high thermal stability, making them potential candidates for photovoltaic material.^[^ ^85^, ^147^ ^]^ Yu et al. introduced the Bi_2_O_2_S nanocrystals into a binary polymer solar cell (PSC) consisting of poly[[4,8‐bis[(2‐ethylhexyl)oxy] benzo [1,2‐b:4,5‐b′] dithiophene‐2,6‐diyl] [3‐fluoro‐2‐(2‐ethylhexy) carbonyl] thieno [3,4‐b] thiophenediyl]] (PTB7) and [6,6]‐pheny C71‐butyric acid methy ester (PC_71_BM), improving the power conversion efficiency (PCE) from 8.79% to 10.71%.^[^ ^147^ ^]^ This improvement was attributed to the broadened absorption range of sunlight and enhanced crystallinity of PTB7, as it contributed to more efficient photon harvesting and enhanced exciton dissociation. In addition to Bi_2_O_2_S, Yu's team also applied the 2D Bi_2_O_2_Se nanoflakes as an additive in the photosensitive layer of PSCs.^[^ ^85^ ^]^ The 2D Bi_2_O_2_Se nanoflakes in the photosensitive layer formed a new donor/acceptor interface for efficient charge transfer and facilitated crystallization due to the suitable energy level between the PBDB‐T and ITIC. As a result, the PCE of PSCs based on 2D Bi_2_O_2_Se nanoflakes increased from 10.09% to 12.22% due to the promotion of exciton separation and charge transfer in the photosensitive layer. Furthermore, BiOI thin films grown by CVT have also investigated their photovoltaic performance through theory and experiment.^[^ ^148^ ^]^ Hoye et al. predicted BiOI to be tolerant to antisite and vacancy defects according to computations, owing to high defect‐formation energies and a high dielectric constant. Experimental results demonstrated negligible hysteresis, high external quantum efficiency (80%), and higher PCE of the all‐inorganic solar cells (ITO|NiO_x_|BiOI|ZnO|Al).

##### Optical Gas Sensors

**Figure 12:**
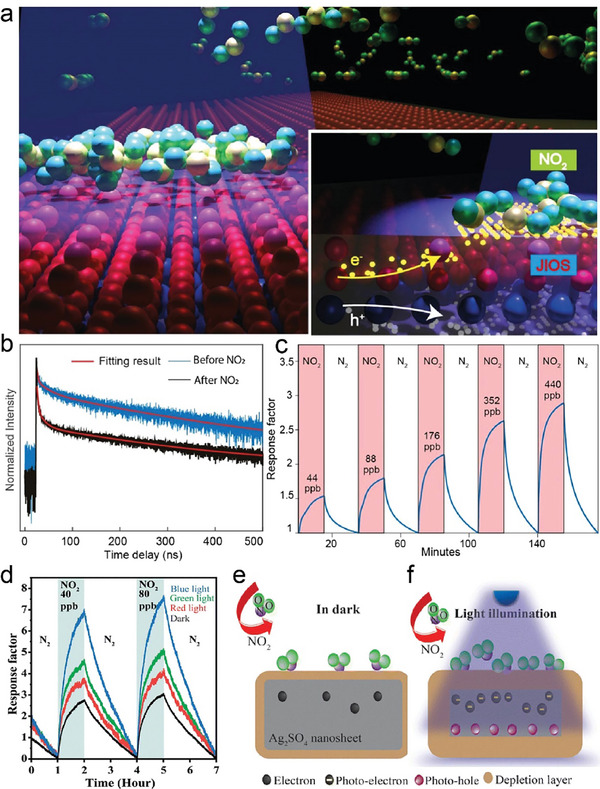
a) Schematic diagram of the adsorption‐based gas sensing mechanism, in which the green‐yellow‐colored NO_2_ gas molecules are accumulating on the light spot of the incident light beam upon the Janus indium oxysulfide (JIOS) lattice. The inset demonstrates the cross‐section of the JIOS crystals and depicts a charge‐carrier separation process with the electrons (yellow dots) and holes (white dots). b) In situ PL lifetime changes before and after NO_2_ gas exposure. The former (blue) has a longer decay component than the latter (black). c) Dynamic sensing performance of JIOS flakes toward NO_2_ gas at concentrations ranging from 44 to 440 ppb under blue light irradiation at room temperature. Reproduced from permission.^[^ ^150b^ ^]^ Copyright 2021, Elsevier. d) Sensing performance of the 2D Ag_2_SO_4_ nanosheets toward NO_2_ gas with concentrations of 40 and 80 ppb under dark conditions and under red‐, green‐, and blue‐light illumination at room temperature. Schematics of the NO_2_ gas‐sensing mechanism at room temperature (e) in the dark and (f) under light illumination. Reproduced from permission.^[^ ^78^ ^]^ Copyright 2022, Royal Society of Chemistry.

Gas sensors are designed to detect and measure the presence and concentration of specific gases in the surrounding environment. The development of gas sensors with high sensitivity, selectivity, and low power consumption has consistently been the focus of researchers.^[^ ^149^ ^]^ The layered mixed‐anion oxycompounds for optical gas sensors are semiconductors. 2D metal oxychalcogenides with larger surface area, abundant active adsorption sites, and longer visible‐light‐driven exciton lifetime make them candidates with strong potential in realizing ultrasensitive gas sensors even at room temperature.^[^ ^79^, ^80^, ^81^, ^82^, ^150^ ^]^ Recently, free‐standing ultra‐thin Jannus indium oxysulfides were prepared through mechanical exfoliation from non‐layered In_2_S_3_.^[^ ^150b^ ^]^ In this unique Janus structure, a tensile strain between the pure sulfide and oxysulfide phases formed due to the crystal coordination mismatching, elevating the lifetime of visible‐light‐driven excitons by over two orders of magnitude compared to that of pure parental In_2_S_3_. Ultrasensitive optoelectronic gas sensors were developed based on this enhanced excitonic interaction and the device exhibited an exceptionally low detection limit of NO_2_ gas down to sub‐ppb level at room temperature, as shown in **Figure** **12**a–c. In a similar approach, Ou's group synthesized ultrathin 2D Ag_2_SO_4_ nanosheets from non‐layered Ag_2_S. The material was subsequently characterized, revealing a bandgap of 1.87 eV and an extended exciton lifetime of 2.06 ns. It was then utilized in investigations to explore its gas sensing capabilities.^[^ ^78^ ^]^ As shown in Figure 12d‐f, the developed sensors exhibited light‐dependent responses to NO_2_ gas with the highest response factor of 8.39% for 160 ppb NO_2_ under blue‐light illumination at room temperature. Another 2D oxyselenide semiconductor, Bi_2_O_2_Se nanoplates were also successfully applied for a resistive oxygen sensor with a detection limit of 0.25 ppm and long‐term stability at room temperature.^[^ ^151^ ^]^ Such remarkable gas performance could be ascribed to the abundant active adsorption sites of amorphous Se atoms and larger specific surface area, which were confirmed by scanning tunneling microscopy and first‐principle calculations.

### 4.2 Catalytic properties and applications

#### 4.2.1 . Catalytic properties

**Figure 13:**
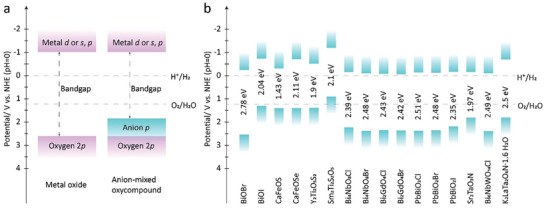
(a) Schematic band structures for a metal oxide and an anion‐mixed oxycompound. (b) Schematic valence and conduction band structures for layered anion‐mixed oxycompounds.

Catalytic properties refer to a material's ability to accelerate chemical reactions by lowering the activation energy without being consumed in the process. Layered anion‐mixed materials, featuring unique lamellar structures and narrower band structures, exhibit advanced semiconductor properties, enabling efficient visible‐light‐driven photocatalysis for water splitting, dye degradation, nitrogen fixation, and electrocatalysis for CO_2_ reduction. In detail, layered oxychalcogenides, oxyhalides, and oxynitrides have been widely applied in photocatalytic water splitting. As shown in **Figure** **13**a, they have narrower bandgaps compared with their oxides’ counterpart. This is because anions less electronegative than oxygen exhibit higher energy levels, resulting in a more negative valence band and consequently reducing the bandgap. The narrower bandgap makes them suitable for visible‐light absorption.^[^ ^3^, ^30^, ^56^, ^152^ ^]^ The bandgap positions of layered anion‐mixed materials applied for photocatalytic water splitting have been summarized in Figure 13b. It is noteworthy that some of oxyhalides possess unusual oxygen dispersive, enabling them resistant to photocatalytic self‐oxidative decompositions.^[^ ^3b,c^ ^]^ In addition, layered structures exhibit 2D charge transport properties and charge recombination prohibition, promoting efficient charge transport. Due to their unique layered structure, layered bismuth oxyhalides can form low‐dimensional structures with different exposed facets, oxygen vacancies, isolated single metal atoms doping, and van der Waals gaps (VDWGs), enabling them to be highly efficient catalysts in dye degradation, nitrogen fixation, and CO_2_ reduction.

#### 4.2.2 . Applications

##### Water splitting

With the goal of realizing bulk production of hydrogen from renewable sources, photocatalytic water splitting is a promising approach that has attracted intensive research interest. The process is accomplished when holes and electrons are used for water oxidation and reduction, respectively, which is known as oxygen evolution reaction (OER) and hydrogen evolution reaction (HER). Layered multi‐anion materials have narrower bandgaps compared to their oxide counterparts, which allow the harvest of visible photons for overall water splitting. In the meantime, ideal photocatalysts are expected to have a suitable thermodynamic potential, be reproducible and stable against photocorrosion. In this regard, layered bismuth oxyhalides and oxysulfides semiconductors are the star materials to feed this need, thanks to their strong light absorption ability and tunable optical properties.^[^ ^3^, ^5^, ^53^, ^56^, ^152^ ^]^

Metal oxides with bandgaps greater than 3 eV are not suitable for overall water splitting in the visible‐light region (400–700 nm). Some metal sulfides have narrower band structures, but the S^2−^ and Se^2−^ anions are more susceptible to oxidation than water which leads to an imbalanced production ratio of H_2_ and O_2_ during water splitting.^[^ ^153^ ^]^ Ternary oxysulfides, Bi_2_O_2_Se, a new material discovered in recent years, was investigated for its photocatalytic hydrogen evolution as a co‐catalyst.^[^ ^154^ ^]^ Remarkably, the combination of TiO_2_ led to significant enhancement in H_2_ evolution, reaching a rate of 1240.7 µmol g^−1^ h^−1^ under UV light. In comparison, pure Bi_2_O_2_Se did not facilitate hydrogen production, and pure TiO_2_ alone achieved a hydrogen production rate of merely 21 µmol g^−1^ h^−1^. Such impressive improvement in performance is possibly attributed to the suppression of the recombination of the photogenerated electron‐hole in Bi_2_O_2_Se/TiO_2_ nanostructures. In another case, the tightly bonded 2D‐2D heterojunction materials consisting of Bi_2_WO_6_ and Bi_2_O_2_S were developed by the presence of a similar structural unit of a^[^Bi_2_O_2_]^2+^ slice within their crystal structures.^[^ ^152^ ^]^ The judicious combination of Bi_2_WO_6_ and Bi_2_O_2_S through a 2D‐2D heterojunction not only extends light absorption in the visible range but also significantly enhances photo(electro)chemical water splitting efficiencies compared to that of bare Bi_2_WO_6_ nanosheets alone, which is due to the close‐bonding‐promoted interfacial charge separation. In addition, quaternary oxysulfides are also a group of potential candidates for photocatalysts. Layered Sm_2_Ti_2_S_2_O_5_ as a stable photocatalyst was first applied for the evolution of H_2_ or O_2_. Domen et al. improved the photocatalytic activities for overall water splitting by tuning the preparation conditions of Sm_2_Ti_2_S_2_O_5_.^[^ ^52^ ^]^ An appropriate sulfurization temperature contributes to good crystallinity and high photocatalytic activities. Later, they discussed the relationship between the photocatalytic activity and the band structure of the series Ln_2_Ti_2_S_2_O_5_ (Ln = Pr, Nd, Sm, Gd, Tb, Dy, Ho, and Er). As the valence band position of [Gd‐Er]_2_Ti_2_S_2_O_5_ is thermodynamically unfavorable, they have lower photocatalytic activity for the oxidation of H_2_O to O_2_.^[^ ^96^ ^]^ Among the prepared [Gd‐Er]_2_Ti_2_S_2_O_5_ products, Sm_2_Ti_2_S_2_O_5_ has the highest performance for O_2_ evolution with a quantum efficiency of 0.7%. Domen's group conducted further research and found that post‐treatment of Sm_2_Ti_2_S_2_O_5_ through annealing with sulfur and subsequent nitric acid etching would bring significant improvement in the photocatalytic H_2_ evolution rate by a factor of 4.5.^[^ ^155^ ^]^ Besides, with the combination of rutile TiO_2_ and NaI, Pt‐loaded Sm_2_Ti_2_S_2_O_5_ were successfully applied for Z‐scheme water splitting as photocatalysts, which is a key step for efficient solar hydrogen production in Z‐scheme system. However, since TiO_2_ is only active under UV light, this system cannot function under visible‐light. To address this issue, Domen and co‐workers loaded IrO_2_ and Pt on Sm_2_Ti_2_S_2_O_5_, serving as an oxidative cocatalyst and a reductive cocatalyst, respectively. This configuration resulted in visible‐light‐driven Z‐scheme water splitting into H_2_ and O_2_.^[^ ^97^ ^]^ Recently, Domen's group reported another promising photocatalytic material of Y_2_Ti_2_O_5_S_2_, an oxysulfide semiconductor with a bandgap of 1.9 eV, which shows direct water splitting capability under visible‐light.^[^ ^3a^ ^]^

**Figure 14:**
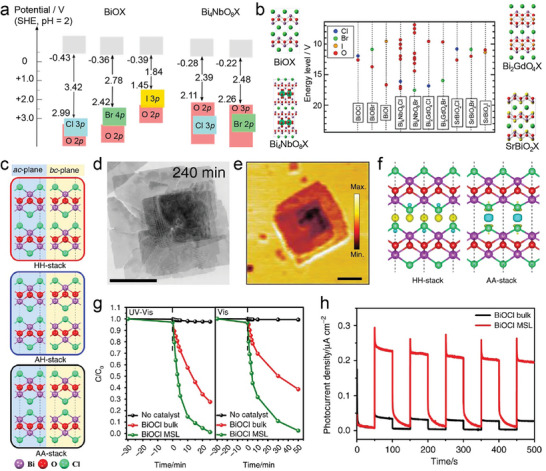
(a) Schematic valence and conduction band structures of BiOX (X = Cl, Br, I) and Bi_4_NbO_8_X (X = Cl, Br). (b) Madelung site potentials of BiOX (X = Cl, Br, I), Bi_4_NbO_8_X (X = Cl, Br), Bi_2_GdO_4_X (X = Cl, Br), and SrBiO_2_X (X = Cl, Br, I). Reproduced with permission.^[^ ^3c^ ^]^ Copyright 2017, American Chemical Society. c) The bilayer structures with different stacking patterns of HH‐stack, AH‐stack, and AA‐stack structures. d) TEM image of BiOCl MSLs (scale bar: 500 nm). e) The near‐field amplitude image from the s‐SNOM measurement shows the distribution of free carriers. f) Charge density differences between the bilayers (in AA‐stack) of BiOCl MSL and individual monolayers (in HH‐stack) of BiOCl bulk. g) The photocatalytic degradation rate of Rhodamine B versus time without catalyst and with BiOCl bulk, BiOCl MSL nanosheets as catalysts. h) Transient photocurrent response versus time without bias, measured in a 0.5 mol  L^−1^ Na_2_SO_4_ aqueous solution and the Ag/AgCl reference electrode under visible‐light irradiation. Reproduced with permission.^[^ ^74^ ^]^ Copyright 2019, Springer Nature.

The presence of additional halide anions facilitates the generation of suitable band positions and gaps, making them favorable for a wide range of photocatalytic reactions. Most research focuses on the Bi‐containing oxyhalides covering ternary, quaternary, and quinary compounds. BiOI, with a bandgap of 2.04 eV, is a ternary oxyhalide photocatalyst for water splitting under visible light.^[^ ^156^ ^]^ Due to S^2−^ and N^3−^ being less electronegative than O^2−^, layered oxysulfides and oxynitrides are generally unstable, making them unsuitable for water oxidation applications. However, quaternary oxyhalides such as Sillén‐Aurivillius perovskite Bi_4_NbO_8_Cl can absorb visible light and exhibit high stability against water oxidation.^[^ ^3b^ ^]^ In comparison with 3.5 eV bandgap of BiOCl, Bi_4_NbO_8_Cl exhibits a relatively small value of 2.4 eV because the VBM of Bi_4_NbO_8_Cl is unusually high. According to the DFT calculations of band structure, this remarkable stability can be attributed to the highly dispersive O 2*p* orbitals rather than the Cl 3*p* orbitals. The schematic band structures of BiOX (X = Cl, Br, I) and Bi_4_NbO_8_X (X = Cl, Br) are shown in **Figure** **14**a. The result contrasts with the band structure of oxynitrides, where anions other than oxygen constitute VBM. The origin of this special structure was investigated by Kato and co‐workers.^[^ ^3c^ ^]^ Through a systematic investigation of the band structures of four series of layered bismuth oxyhalides, combining experimental data with theoretical analysis, it was found that the variations in their valence‐band structures can largely be explained by differences in the Madelung energy at the oxygen site (Figure 14b). The oxygen anion in fluorite‐like blocks, such as the [Bi_2_O_2_] slab in Bi_4_NbO_8_Cl, causes an upward shift in the valence band, with the degree of electrostatic destabilization varying based on the composition and arrangement of the layers. This study indicates that Madelung analysis can be used to predict and design the valence band structures of bismuth and other layered oxyhalides, even in compounds where performing DFT calculations is challenging. Additionally, a quinary oxyhalide Bi_6_NbWO_14_Cl possessing a Sillén‐Aurivillius‐related structure was applied for oxygen evolution in Z‐scheme water splitting under visible light.^[^ ^35^ ^]^ These studies further emphasize the significance of the Sillén–Aurivillius framework, suggesting that bandgap tuning can be achieved by altering structural motifs and their sequence in Sillén‐Aurivillius‐related systems.

Layered oxynitrides, primarily composed of quaternary and quinary compounds, have shown great potential as photocatalysts for water splitting. This is attributed to the lower electronegativity of the N^3−^ anion, which results in a more negative valence band position and a narrower bandgap compared to conventional single‐anion oxides, thereby facilitating visible light absorption. Non‐layered perovskite oxynitrides, such as SrTaO_2_N and CaNbO_2_N, suffer from poor photocatalytic activity, due to the self‐oxidative decompositions even with a hole scavenger present.^[^ ^157^ ^]^ However, quaternary oxynitrides like Sr_2_TaO_3_N were obtained by adding extra SrO layers into SrTaO_2_N, showing enhanced light adsorption.^[^ ^30^ ^]^ Notably, Sr_2_TaO_3_N can facilitate photocatalytic water oxidation under visible light without a cocatalyst and minimize self‐oxidative decomposition which is often observed in SrTaO_2_N. This is mainly attributed to additional SrO layers which help shift the valence band edge and stabilize N species. Theoretical calculations by Pauling's second rule suggest that Sr_2_TaO_3_N exhibits typical 2D charge transport properties, with the CBM and VBM located within TaN_2_O_2_ square planes, promoting efficient charge transport. Quinary oxynitrides like NaLaTiO_4–x_N_y_, with more diverse types of cations, exhibit unique structural characteristics essential for nitrogen uptake, defect concentration, and microstructures.^[^ ^36^ ^]^ For instance, NaLaTiO_4–x_N_y_ demonstrates excellent photocatalytic performance for water reduction and oxidation, with an apparent quantum efficiency of ≈3.2% at 420 ± 20 nm, surpassing many other metal oxynitride photocatalysts. Theoretical studies indicate that Na/La ordering induces a strong electric field across Ti(O, N)_6_ octahedral layers, disrupting the alignment of molecular orbitals, which helps prevent intralayer charge recombination. While layered oxynitrides are often sensitive to moisture and can undergo hydrolysis with nitrogen loss, leading to reduced visible‐light absorption, recent advancements have mitigated this issue.^[^ ^158^ ^]^ For example, Maeda's group synthesized a novel layered perovskite oxynitride, K_2_LaTa_2_O_6_N which exhibits high water tolerance and performs well as a photocatalyst for hydrogen production under visible light.^[^ ^1f^ ^]^ This material retained its visible‐light‐absorption capability during an in situ H^+^/K^+^ exchange process in an aqueous solution. When protonated and modified with an iridium (Ir) cocatalyst, K_2_LaTa_2_O_6_N demonstrated superior catalytic activity for hydrogen evolution under visible light using iodide (I^−^) as an electron donor. Its performance was six times greater than one of the leading oxynitride photocatalysts, Pt/ZrO_2_/TaON. Moreover, overall water splitting was achieved using Ir‐loaded, protonated K_2_LaTa_2_O_6_N in combination with Cs‐modified Pt/WO_3_ as an oxygen evolution photocatalyst, facilitated by an I_3_^−^/I^−^ shuttle redox couple. The structure of materials significantly impacts their photocatalytic activity. A comparison of the hydrogen evolution activity between K_2_Ca_2_Ta_3_O_9_N·2H_2_O, which has a three‐layer Ruddlesden‐Popper structure, and K_2_LaTa_2_O_6_N·1.6H_2_O, with a two‐layer Ruddlesden‐Popper structure.^[^ ^159^ ^]^ Their findings suggest that the two‐layer perovskite oxynitride is more effective for visible‐light photocatalysis. This contrasts with the behaviors of layered metal oxides, where three‐layer oxides generally demonstrate much higher photocatalytic activity.

##### Dye Degradation

Photocatalytic dye degradation in aqueous solution is increasingly important given the prevalence of organic pollution. Low‐dimensional oxyhalides with facet‐dependent photoreactivity are potential photocatalysts for the degradation of organic dyes. Zhang et al. prepared the BiOCl single‐crystalline nanosheets with exposed {001} and {010} facets.^[^ ^102^ ^]^ The obtained exposed {001} facets exhibited higher activity for direct semiconductor photoexcitation pollutant degradation under UV light, which is because of a cooperative effect between the surface atomic structure and suitable internal electric fields. Conversely, the nanosheets with exposed {010} facets possessed superior activity for indirect dye photosensitization degradation under visible light, owing to their larger surface area and open channel characteristics. Another Zhang and teams developed a solvothermal method to selectively synthesize BiOBr nanosheets with atomic thickness, prominently exposing the active (001) facets. This strategy optimizes the band structure, enhancing electron‐hole pair separation and significantly improving visible‐light photocatalytic activity.^[^ ^4^ ^]^ Hao's group^[^ ^103^ ^]^ illustrated experimentally and theoretically that the photocatalytic performance of BiOBr nanosheets can be tuned by the inner strain effect, leading to ≈100% efficiency in rhodamine B degradation within 30 min. Chen et al. demonstrated FeOCl nanosheets and plates obtained by liquid exfoliation. The FeOCl nanosheets exhibited superior catalytic performance in degradation of phenol compared to its plates form at room temperature, under neutral pH conditions and sunlight irradiation.^[^ ^83^ ^]^ Furthermore, MSLs in bismuth oxychloride spiral nanosheets have been synthesized using a bottom‐up strategy,^[^ ^74^ ^]^ forming a crystal structure with three bilayer structures and different stacking patterns, including HH‐stack, AH‐stack, and AA‐stack (Figure 14c‐f). The HH‐stack pattern represents the ground‐state bulk BiOCl structure, inducing a 0.55 eV bandgap reduction. Meanwhile, the AA‐stack structure exhibits a significant charge depletion between head‐to‐head Cl atoms from adjacent layers, indicating a potent interlayer anti‐bonding interaction. Hole carriers localized at the AA‐stack region in MSL BiOCl lead to improve the transfer of carriers, thereby enhancing the photocatalytic degradation of Rhodamine B by MSL BiOCl spiral nanosheets (Figure 14g and h).

##### Nitrogen Fixation

The Haber‐Bosch process for nitrogen fixation into ammonia (NH_3_) needs to use the catalyst under harsh conditions (400–450 °C at 150–200 atm) due to the robust stability of N_2_.^[^ ^160^ ^]^ Such demanding operational parameters cause substantial energy consumption, which in turn increases the cost associated with NH_3_ manufacturing and brings a negative impact on the environment. To overcome the obstacle, researchers have turned to the photocatalytic NH_3_ synthesis process, which has gained significant attention in recent years for its energy and environmental benefits.^[^ ^161^ ^]^ This innovative process employs cost‐effective semiconductors as catalysts to transform N_2_ and H_2_O to NH_3_ driven by light photoirradiation under much milder conditions – room temperature and ambient pressure.^[^ ^162^ ^]^

Bismuth oxyhalide with abundant oxygen vacancies serves as another promising candidate. It showcases high N_2_ adsorption capability and efficient separation of photoexcited charge carriers, demonstrating excellent nitrogen fixation performance in the absence of any noble metal cocatalysts or metal complexes.^[^ ^163^ ^]^ In 2015, Li et al.^[^ ^164^ ^]^ synthesized BiOBr nanosheets with surface oxygen vacancies on the exposed {010} facets by solvothermal method, which demonstrated efficient N_2_ fixation performance with the NH_3_ yield of 104.2 µmol h^−1^ g^−1^ under visible‐light (λ > 420 nm) at 25 °C. In comparison, the reference BiOBr nanosheets without oxygen vacancies show poor efficiency for NH_3_ production. In situ diffuse reflectance Fourier transform infrared (FTIR) spectroscopy of BiOBr with surface oxygen vacancies reveals a gradual increase of several adsorption bonds, which are attributed to the ν (N−H) stretching mode, ν (O−H) stretching mode, σ (N─H) bending mode, and σ (O─H) bending mode, respectively. Interestingly, no obvious change was observed in the FTIR spectra of reference BiOBr nanosheets. These findings suggested that the surface oxygen vacancies can considerably increase the N_2_ fixation capability of BiOBr in visible light. In addition to BiOBr, Bi_5_O_7_Br, and Bi_3_O_4_Br have also been identified as promising visible‐light‐responsive bismuth oxybromide catalysts for photocatalytic N_2_ fixation. Wang et al.^[^ ^163b^ ^]^ reported Bi_5_O_7_Br nanotubes, featuring a uniform diameter of 5 nm, which were synthesized through a water‐assisted self‐assembly method. Such Bi_5_O_7_Br nanotubes exhibit strong visible‐light‐driven photo‐catalytic N_2_ fixation ability with a 1.4 mmol h^−1^ g^−1^ NH_3_ production rate in water. Under monochromatic light irradiation at 420 nm, the apparent quantum efficiency was determined to be as high as 2.3%. Recently, Di et al. prepared defect‐rich, single‐unit‐cell Bi_3_O_4_Br layers by a PVP self‐assembly strategy.^[^ ^165^ ^]^ While the defect‐deficient Bi_3_O_4_Br flakes were synthesized through an additional hydrothermal treatment of as‐synthesized Bi_3_O_4_Br layers. Photocatalytic investigation revealed that defect‐Bi_3_O_4_Br layers display state‐of‐the‐art activity for NH_3_ production with 380.0 µmol g^−1^ h^−1^, much higher than that of defect‐deficient Bi_3_O_4_Br flakes. Apart from bismuth oxybromide, bismuth oxychloride, and bismuth oxyiodide were also exploited for nitrogen fixation. For instance, interstitial carbon‐doped BiOI was synthesized via a hydrothermal method and exhibited a photocatalytic nitrogen fixation rate of 311 µmol g^−1^ h^−1^, which was 3.7 times higher than that of pure BiOI.^[^ ^166^ ^]^

##### CO_2_ Reduction

Layered bismuth oxyhalides BiOX (X = Cl, Br, I) are impressive catalysts for CO_2_ reduction due to their appealing energy band structures, layered structure, and chemical stability.^[^ ^167^ ^]^ In an electrochemical CO_2_ reduction reaction, bismuth oxyiodides with different chemical compositions could influence electrocatalytic performances. A higher amount of bismuth and iodine can contribute to an outstanding Faradaic efficiency of 89 % and a higher partial current density in formate production.^[^ ^168^ ^]^

**Figure 15:**
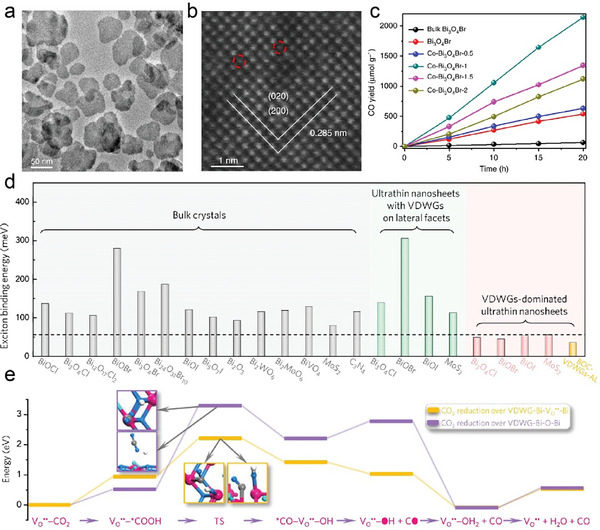
a) TEM image and (b) Atomic resolution HAADF‐STEM images of Co‐Bi_3_O_4_Br. c) Photoreduction of CO_2_ into CO over Bi_3_O_4_Br and Co‐Bi_3_O_4_Br materials. CO_2_ adsorption isotherms of Bi_3_O_4_Br and Co‐Bi_3_O_4_Br. Reproduced with permission.^[^ ^165^ ^]^ Copyright 2019, Springer Nature. d) Comparisons of exciton binding energy (E_b_) of BOC‐VDWGs‐AL and some representative layered photocatalysts. e) Gibbs free energy diagrams of intermediates generated during CO_2_‐to‐CO catalysis over VDWGs‐Bi‐O‐Bi and VDWGs‐Bi‐VO^••^‐Bi. Reproduced with permission.^[^ ^6^ ^]^ Copyright 2021, Springer Nature.

Although the catalyst of layered BiOX materials has successfully realized CO_2_ reduction, the high surface charge recombination rate and the lack of sufficient surface‐active sites limit their catalytic performance. Several strategies were employed to boost their catalytic behavior, including the incorporation of isolated single metal atoms into 2D ultrathin nanosheets, the creation of oxygen defects, and the introduction of VDWGs.^[^ ^6^, ^165^, ^169^ ^]^ Besides, single‐metal‐atom catalysts, possessing the smallest size and highest metal dispersity, offer an increase in active sites and maximum atomic utilization efficiency. These characteristics display significant potential in catalytic applications. Liu et al. successfully prepared isolated single‐atom cobalt in Bi_3_O_4_Br atomic layers (Co‐Bi_3_O_4_Br), as shown in **Figure** **15**a,b.^[^ ^165^ ^]^ The introduction of cobalt single atoms in the ultrathin Bi_3_O_4_Br improved the charge transition, charge‐carrier separation kinetics, CO_2_ adsorption, and activation. Thus, the CO_2_ activation energy barrier decreased, and the step of CO_2_ formation was optimized. As shown in Figure 15c, an exceptional and high selective yield of CO at 107.1 µmol g^−1^ h^−1^ was acquired over the Co‐Bi_3_O_4_Br, which was roughly 32 and 4 times higher than that of bulk Bi_3_O_4_Br and Bi_3_O_4_Br atomic layer, respectively.

Moreover, the implantation of single‐atom Fe in Bi_4_O_5_I_2_ was also investigated. Experimental results showed that Fe implantation can decrease the surface work function and improve the charge transition rate.^[^ ^169a^ ^]^ Benefiting from the single‐atom Fe introduction, the optimized Bi_4_O_5_I_2_‐Fe_30_ exhibited a CO and CH_4_ evolution rate of 23.77 and 4.98 µmol g^−1^ h^−1^, respectively, which is ≈5 times higher than that of Bi_4_O_5_I_2_ alone. However, insufficient active sites remain a significant limitation for the overall conversion efficiency of solar‐driven CO_2_ reduction. To overcome this issue, the creation of surface oxygen defects can enhance CO_2_ photoreduction by leveraging abundant dangling bonds. Liu and team have prepared the defect‐rich Bi_12_O_17_Cl_2_ nanotubes with a bilayer thick tube wall.^[^ ^169b^ ^]^ Compared to the bulk counterparts, Bi_12_O_17_C_l2_ nanotubes can perform photocatalytic CO_2_ reduction with a higher CO formation rate of 48.6 µmol g^−1^ h^−1^ in the absence of sacrificial reagent and cocatalyst (≈16.8 times enhancement). In addition, rich VDWGs can also contribute to enhancing the photocatalytic reduction of CO_2_. In another example, Shi et al. have synthesized sub‐3 nm thick BiOCl nanosheets with VDWGs on 2D facets and oxygen vacancies by pH adjustment and C doping.^[^ ^6^ ^]^ The VDWGs‐rich BiOCl atomic layers (BOC‐VDWGs‐AL) demonstrate a high photocatalytic CO_2_‐to‐CO conversation rate of 188.2 µmol g^−1^ h^−1^ in water without any hole scavenger or cocatalyst. This high performance can be attributed to a weak excitonic confinement power of 36 meV, which is much lower than those of other layered photocatalysts (Figure 15d).^[^ ^6^ ^]^ In the meantime, the creation of VDWGs‐Bi‐VO••‐Bi (VO••: oxygen vacancies) defects enhances the CO_2_‐to‐CO transformation through the simultaneous optimization of CO_2_ activation, *COOH splitting, and *CO desorption (*: intermediates). As shown in Figure 15e, the transition states linking *COOH to *CO + *OH exhibit the largest energy barriers during both reaction pathways. It is clear that the in situ formation of VO•• promotes *COOH cleavage as the energy barrier of VDWGs‐Bi‐VO••‐Bi (1.27 eV) is 1.51 eV lower than that of VDWGs‐Bi‐O‐Bi (2.78 eV). Besides, it is discovered that VO•• promotes direct *CO desorption (−0.39 eV) instead of hydrogenation to generate *HCO species (+0.25 eV). Additionally, the *CO CO step is endothermal for VDWGs‐Bi‐O‐Bi compared to exothermal for VDWGs‐Bi‐VO••‐Bi. Finally, compared to VDWGs‐Bi‐O‐Bi, VDWGs‐Bi‐VO••‐Bi shows stronger adsorption capability.

### 4.3 Thermoelectric Properties

Thermoelectric properties refer to the ability of materials to convert thermal energy into electrical energy under a temperature gradient. The high performance of thermoelectric materials is determined by the dimensionless thermoelectric *ZT*, which is associated with the high Seebeck coefficient (*S*) and electrical conductivity (σ), as well as the low electronic (κ_e_) and lattice (κ_l_) contributions to thermal conductivity (κ), according to the Equation (1)^[^ ^170^ ^]^:

(1) $$ZT=\frac{S^{2}\sigma }{\kappa_{e}+\kappa_{l}}\;T$$

In general, layered structures are advantageous for thermoelectric applications, primarily due to their inherent phonon anharmonicity, which reduces lattice thermal conductivity. Additionally, the reduced dimensionality in layered structures can enhance electronic properties through quantum confinement effects. Among layered multi‐anion oxygenated materials, layered oxychalcogenides compounds are the main family with notable thermoelectric properties. This is attributed to their uniquely stacked alternating conducting (MCh) and insulating (MO) layers, leading to intrinsically low thermal conductivity and other interesting electronic properties.^[^ ^171^ ^]^

**Figure 16:**
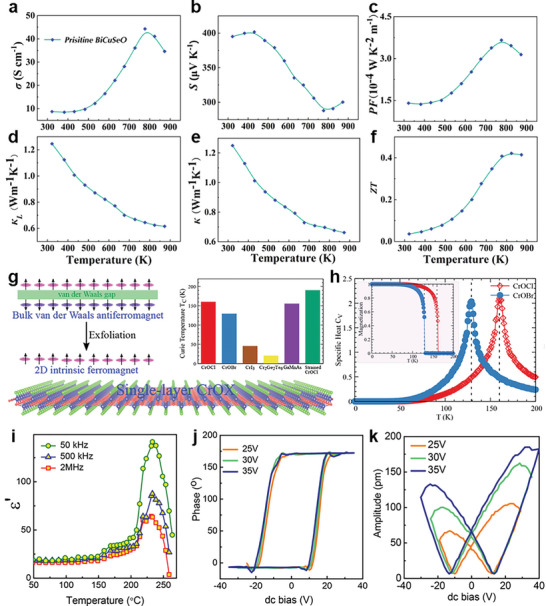
a) Electrical conductivity, σ, b) Seebeck coefficient, *S*, c) Power factor, PF, d) Lattice thermal conductivity, κ_l_, e) Total thermal conductivity, κ, f) Dimensionless figure‐of‐merit, *ZT* of BiCuOSe. Reproduced with permission.^[^ ^172^ ^]^ Copyright 2023, Elsevier. g) Schematic of magnetic properties of CrOX in bulk and monolayer. Inset of (g) is Curie temperature in comparison with 2D CrI_3_ (45 K), Cr_2_Ge_2_Te_6_ (20 K), GaMnAs (155 K), and single‐layer CrOCl under 5% biaxial tensile strain. h) Specific heat C_V_ respective to temperature for the CrOCl and CrOBr monolayers and the inset shows the corresponding magnization. The magnetic transition temperatures *T*_C_ are denoted by dashed lines. Reproduced with permission.^[^ ^186^ ^]^ Copyright 2018, American Chemical Society. i) Temperature variation of the real part of relative permittivity (ε′) at different frequencies for Bi_2_O_2_Se nanosheet. “Off‐state” (j) phase and (k) amplitude of SS‐PFM signal from Bi_2_O_2_Se nanosheet at room temperature exhibiting ferroelectricity with 180° polarization switching. Reproduced with permission.^[^ ^181^ ^]^ Copyright 2019, American Chemical Society.

The combination of p‐ and n‐type thermoelectric materials is crucial for constructing a thermoelectric module, enabling the optimal utilization of the Seebeck effect. Notable p‐type oxychalcogenides include BiCuChO (Ch = S, Se, Te), BiAgChO, Sr_2_AM_2_O_2_Ch_2_ (A = Co, Mn, Zn; M = Cu, Ag; Ch = S, Se), and Bi_2_YO_4_Cu_2_Se_2_. Among these, BiCuChO, particularly BiCuSeO, is extensively studied due to its high Seebeck coefficient, low lattice thermal conductivity, and excellent performance.^[^ ^172^ ^]^ Its thermoelectric‐related parameters are shown in **Figure** **16**a–f. BiCuSeO displays anisotropic thermal and electric conductivity due to its inherent layered structure, with higher conductivity along the in‐plane direction compared to the out‐of‐plane direction, while the Seebeck coefficient remains isotropic.^[^ ^172^ ^]^ Researchers have attempted many strategies including ball milling, doping, and introducing vacancies to enhance the *ZT* of BiCuSeO bulk material.^[^ ^28^, ^173^ ^]^ For example, Liu et al.^[^ ^28^ ^]^ reported a single‐phased BiCuSeO polycrystal via a simple ball milling process, which refined materials’ grains, reduced the lattice's thermal conductivity and enhanced electrical conductivity achieving a *ZT* value of ≈0.7 at 770 K for pristine BiCuSeO. Later, Zhao and the team achieved a high *ZT* of ≈1.4 at 923 K for textured Bi_0.875_Ba_0.125_CuSeO prepared through a hot‐forging process, which doubled the carrier mobility due to textured microstructure and preferred grain orientation.^44^ BiCuSeO also showed a significant absolute *ZT* (≈1.5 at 873 K) across the mid‐to‐high temperature range (≈650–923 K).^[^ ^174^ ^]^ In another case, substituting Cu with Ag in BiCuSeO to create BiAgChO has been predicted through first‐principles calculations to be a promising thermoelectric material due to its high electrical conductivity and Seebeck coefficient.^[^ ^175^ ^]^ The maximum power factors for p‐type BiAgChO could reach up to 25 mW mK^−2^ at 500 K, permitting an excellent thermoelectric material within the mid‐ to high‐temperature range. Beyond these typical thermoelectrical materials, compounds like BiCuChO, BiAgChO, and Sr_2_AM_2_O_2_Ch_2_, which possesses various metal ions, also belong to layered oxychalcogenides. Layered Sr_2_AM_2_O_2_Ch_2_ exhibits low carrier concentration and low electrical conductivity of 10^−8^ S cm^−1^ at room temperature due to its low carrier concentration. In this case, doping cations is the traditional method used to increase the number of holes. Bi_2_YO_4_Cu_2_Se_2_, a metallic variant derived from BiCuSeO, exhibits p‐type metallic conductivity due to the introduction of a single deficient electron (or an additional hole) in the Cu_2_Se_2_ layer from the Bi_2_YO_4_ charge reservoir. This material demonstrates an electrical conductivity of 3 × 10^5^ Ω^−1^ m^−1^ and a Seebeck coefficient of 25 µV K^−1^ at 300 K, highlighting its metallic nature. However, its relatively high thermal conductivity (≈5 W m^−1^ K^−1^) leads to a low maximum *ZT* of ≈0.012 at 300 K.^[^ ^176^ ^]^

Recently, n‐type layered thermoelectric analogues serve as the alternatives to p‐type materials, which have traditionally dominated the field. For example, an n‐type semiconductor Bi_2_O_2_Se initially demonstrated a *ZT* of 0.007 at 300 K by Ruleova et al,^[^ ^177^ ^]^ bismuth deficiency was later demonstrated to enhance its *ZT*.^[^ ^178^ ^]^ Additionally, Sn doping at the bismuth site in a form of Bi_2‐x_Sn_x_O_2_Se further improves its thermoelectric property, achieving a *ZT* value of 0.20 at 773 K.^[^ ^178^, ^179^ ^]^ Another promising material is the oxy‐telluride Bi_2_O_2_Te, a semiconductor with a narrow bandgap of ≈0.23 eV and a *ZT* value of 0.13 at 573 K.^[^ ^18^ ^]^

The thermoelectric properties of Bi_2_O_2_Se have also been extensively studied, with a focus on the effects of dimensionality, strain, and doping. As reported by Sun et al, their research combined the DFT with semiclassical Boltzmann transport theory revealed that the reduced dimensionality of the Bi_2_O_2_Se nanosheet significantly improves its thermoelectric performance, leading to a much higher *ZT* value of 3.35 at 800 K than that of bulk Bi_2_O_2_Se. Furthermore, Qiao's group investigated and optimized the thermoelectric transport properties of the Bi_2_O_2_Se monolayer via biaxial strain by first‐principles methods.^[^ ^180^ ^]^ The *ZT* value of p‐type doped Bi_2_O_2_Se monolayer can reach 4.22 at 800 K under 2% tensile strain. Pan and co‐workers have also enhanced the peak *ZT* of Bi_2_O_2_Se nanosheets to 0.5 at 793 K, 0.69 at 773 K, and 0.59 at 773 K, respectively, through strategies including introducing Se vacancies, Sb doping, and the substituting O with Te.^[^ ^75^, ^76^ ^]^

### 4.4 Ferroelectric Properties

Ferroelectrics are crystalline materials that exhibit spontaneous polarization within a specific temperature range. The polarization can adopt multiple orientations, which can be controlled by an applied electric field. Among these materials, three types of 2D oxycompounds with ferroelectric properties are identified based on the anions type.

Bismuth oxychalcogenides, such as Bi_2_O_2_S, Bi_2_O_2_Se, and Bi_2_O_2_Te, represent a class of materials that exhibit not only high carrier mobility but also ferroelectricity and/or ferroelasticity. According to the research conducted by Wu's group, these materials show potential to be integrated with non‐volatile functional memories as advanced nanocircuits at room temperature.^[^ ^11b^ ^]^ The DFT calculation result indicates that Bi_2_O_2_S exhibits both ferroelectric and antiferroelectric properties for bulk form. Particularly, a thin film with an odd number of layers can exhibit multiferroicity with a nonzero in‐plane polarization. This polarization is switchable through ferroelasticity. Bi_2_O_2_Se, as the analog of Bi_2_O_2_S, exhibits both piezoelectricity and ferroelectricity caused by the in‐plane strain. While Bi_2_O_2_Te only possesses intrinsic vertical ferroelectricity. Moreover, a 4.1% biaxial strain in Bi_2_O_2_Se is predicted to generate giant polarizations, with a piezoelectric coefficient 35 times higher than that of MoS_2_ monolayer. Later, the study by Biswas and colleagues provided experimental confirmation of ferroelectricity in Bi_2_O_2_Se nanosheets.^[^ ^181^ ^]^ The relative permittivity of Bi_2_O_2_Se nanosheets reaches a maximum at T*_c_* = 235 °C with independent frequency (Figure 16i), indicating a ferroelectric‐like transition. Hysteresis behaviors were characterized using a piezoresponse force microscopy (PFM), showing a 180° polarization switching in phase (Figure 16j) and a butterfly‐shaped hysteresis loop in amplitude (Figure 16k). These observations further imply the existence of a spontaneous polarization state in 2D Bi_2_O_2_Se.

The second type of 2D oxycompounds with ferroelectric properties is bismuth‐containing oxyhalides in an Aurivillius phase. The spontaneous orthorhombic distortion in the ultrathin nanosheets breaks the local inversion symmetry, thereby resulting in ferroelectricity.^[^ ^182^ ^]^ For example, the Aurivillius phases in Bi_4_MO_8_Cl have shown ferroelectricity with T*_c_* = 765 and 640 K for M = Nb and Ta, respectively.^[^ ^182a^ ^]^

Perovskite oxynitride materials show ferroelectric properties as well when the O and N anion are in a specific arrangement. The RP Ca_3_Nb_2_N_2_O_5_, as an example, has the a^−^a^−^c^+^ octahedral rotations‐induced ferroelectric polarization up to 25 µC cm^−2^ along the in‐plane crystallographic direction due to hybrid improper ferroelectricity.^[^ ^183^ ^]^

Overall, the degrees of freedom of the materials’ structure may offer extra flexibility and control over the particular ferroelectric properties, within the general field of layered anion‐mixed oxycompounds.^[^ ^46^ ^]^

### 4.5 Magnetic Properties

Magnetic materials, especially 2D vdW magnets, demonstrate great potential in the field of spintronics. Traditional 2D magnetic elements and binary materials suffer from limited layer thickness. Introducing magnetic ions, such as Fe, Cr, Co, Mn, and V into layered oxycompounds brings more opportunities for the development of magnetic materials. In this section, the report to date about magnetic properties of layered oxycompounds will be categorized based on the number of elements and discussed.

Currently, the focus of intensive study in the realm of 2D vdW magnets centers on ternary 3*d* transition‐metal oxyhalides, represented as MOX. In this notation, “M” denotes the metal ion (Fe, Cr, V), and “X” indicates the halogen ion (F, Cl, Br, I). These compounds are renowned for their fascinating spin‐Peierls behavior and fall within the classification of antiferromagnetic semiconductors and insulators. For instance, FeOX (X = F, Cl, Br, I) monolayers are predicted to be a magnetic Mott insulator with relatively high Neel temperatures in their antiferromagnetic ground state, using first‐principles calculations and Monte Carlo simulations.^[^ ^118^, ^184^ ^]^ More importantly, biaxial compression strain can raise the expected Néel temperatures of FeOX (X = F, Cl, Br, I) monolayers. The strained FeOF and FeOI monolayers have Néel temperatures (T*_N_*) close to 200 K, indicating stronger antiferromagnets and higher Néel temperatures compared to that of other available 2D magnets.^[^ ^118^ ^]^ Besides theory calculations of 2D FeOX, several experimental investigations on their magnetic properties have been conducted, suggesting the significance of 2D vdW magnet family.^[^ ^1^, ^185^ ^]^ In general, ferromagnetic 2D semiconductors exfoliated from the parental bulk materials will maintain the same ferromagnetic properties. Interestingly, CrOX (CrOCl and CrOBr monolayers) were predicted to exhibit robust ferromagnetic ordering based on ab initio calculations, while their bulk CrOX crystals are antiferromagnetic (Figure 16g).^[^ ^186^ ^]^ This discovery showcases the possibility of obtaining 2D ferromagnet semiconductors from their parental crystals. Furthermore, the Curie temperatures of 2D CrOCl and CrOBr are 160 and 129 K, respectively (Figure 16h). It is noteworthy that the Curie temperature of CrOCl monolayers surpasses the record (155 K) – set by the extensively studied dilute magnetic semiconductor GaMnAs,^[^ ^187^ ^]^ presenting new avenues for future explorations and applications in spintronics at an atomic level.

Quaternary oxychalcogenides with magnetic properties mainly include two types of materials. One is A_2_O_2_Mn_2_OSe_2_ (A = La‐Sm), which is composed of edge‐sharing A_4_O tetrahedra layers separated from unusual anti‐CuO_2_ type [M_2_O]^2+^ layers by a layer of Se^2−^ ions.^[^ ^188^ ^]^ A_2_O_2_Mn_2_OSe_2_ (A = La‐Sm) shows an antiferromagnetic structure at T*_N_* = 162–184  K, which is attributed to a structural distortion caused by the static displacement of oxygen ions out of the square planar [Mn_2_O]^2+^ layer.^[^ ^188a^ ^]^ The other magnetic materials, Na_2_Fe_2_OSe_2_, is constructed by the alternating arrangement of [Fe_2_OSe_2_]^2−^ blocks and double layers of Na^+^ along the *c‐*axis. An antiferromagnetic phase transition at T*_N_* = 73 K was revealed by magnetic susceptibility and heat capacity measurements. A broad maximum in magnetic susceptibility and a gradual decay in specific heat were also observed when the temperature exceeded T*_N_*, indicating the existence of 2D short‐range spin correlations.^[^ ^42d^ ^]^

Quinary oxychalcogenides are known to exhibit antiferromagnetic ordering. Magnetic susceptibility measurements of Sr_2_CoO_2_M_2_Se_2_ (M = Cu or Ag) reveal complex antiferromagnetic transitions.^[^ ^48d^ ^]^ In Sr_2_CoO_2_Cu_2_Se_2_ and Sr_2_CoO_2_Ag_2_Se_2_, a broad maximum of ≈250 and 210 K are observed in magnetic susceptibility, respectively, indicating low‐dimensional antiferromagnetic ordering of the cobalt sublattice. Sr_2_CoO_2_Ag_2_Se_2_ displays a second maximum at a lower temperature of 70 K, resembling materials like La_2_CoO_4_ and Ba_2_CoO_2_Cu_2_S_2_ with successive antiferromagnetic transitions. Additionally, both compounds exhibit crossovers ≈37 K for Sr_2_CoO_2_Cu_2_Se_2_ and 60 K for Sr_2_CoO_2_Ag_2_Se_2_, suggesting a possible antiferromagnetic to spin glass transition. Differences between field‐cooled and zero‐field‐cooled susceptibilities at these temperatures imply spin‐glass freezing of magnetic clusters. The low‐temperature Curie‐like term in Sr_2_CoO_2_Ag_2_Se_2_’s susceptibility is likely brought by paramagnetic impurities. Similarly, Sr_2_MnO_2_M_2‑δ_Se_2_ (M = Cu or Ag) exhibits high‐temperature Curie‐Weiss behavior, followed by low‐temperature antiferromagnetic transitions at 54 and 67 K, respectively.^[^ ^48d^ ^]^ Besides, Ca_4_Fe_2_Cu_2_Ch_2_O_6_ (Ch = S or Se) represents antiferromagnetic ionic semiconductors. These materials are composed of alternating layers: non‐magnetic chalcogenide blocks (Cu_2_Ch_2_) and antiferromagnetic oxide blocks (Ca_4_Fe_2_O_6_). They feature an antiferromagnetic spin configuration within Fe sublattice, highlighting their complex magnetic interactions.^[^ ^48i^ ^]^

Here, the structural and magnetic properties of vdW layered oxycompounds are summarized in **Table** **5**.

**Table 5:** Summary of typical 2D oxycompounds materials‐based photodetectors.

Materials	Responsivity [A/W]	Detectivity [Jones]	Wavelength Range [nm]	Response Time [ms]	Ref.
Bi_2_O_2_S	3.73× 10^−4^@ visible‐light	‐^a)^	Visible‐light	–	[17]
Bi_2_O_2_S	4 @ 785 nm	–	NIR^b)^	100	[143]
Bi_2_O_2_S	0.013 @ 365 nm	2.3 × 10^10^	365–850	10/45	[145]
Bi_2_O_2_S	0.059 @ 532 nm	6.77 × 10^9^	532	81.9	[67]
Bi_9_O_7.5_S_6_	17× 10^−6^ @ visible‐light	–	Visible‐light	50	[20]
Bi_2_O_2_Se	65 @ 1200 nm	3.0 × 10^9^	IR^c)^	≈1 × 10^−9^	[98]
Bi_2_O_2_Se	3.5 × 10^4^ @ 532 nm	9.0 × 10^13^	Visible‐light	0.308/ 0.448	[146]
Bi_2_O_2_Se	6.5	8.3 × 10^11^	NIR	2.8	[59]
Bi_2_O_2_Se	4.11× 10^−6^	–	–	2700/4200	[69]
Bi_2_O_2_Se	75.14 @ 360 nm	3.95 × 10^12^	–	78.8	[67]
Bi_2_O_2_Se	1.17 × 10^5^ @ 532 nm	1.95 × 10^12^	IR	0.6/0.4	[100]
BiOCl	8 @ 266 nm	–	UV^d)^	1.8× 10^−8^	[91]
BiOCl	35.7 @250 nm	2.2 × 10^10^	UV	–	[21]
BiOI	2.6 × 10^−2^ @ 473 nm	8.2 × 10^11^	Visible‐light	120	[23]
In_2_O_3‐x_S_x_	3400 @ UV	2.18 × 10^13^	UV	–	[144]

^a)^ Not mentioned.

^b)^ Near‐infrared.

^c)^ Infrared.

^d)^ Ultraviolet

### 4.6 Others

Layered multi‐anions materials have also been occasionally investigated for some other interesting applications, including intrinsic ferromagnets^[^ ^186^ ^]^ and nonlinear photonic devices.^[^ ^116d^ ^]^ Ultrathin Bi_2_O_2_Se nanosheets (≈2 nm) also showed an ordered ferroelectric ground state at room temperature, as reported by Biswas et al., that the behavior is from the spontaneous orthorhombic distortion and destruction of local inversion symmetry.^[^ ^181^ ^]^ Sun et al. demonstrated intrinsic ferromagnetism of single‐layer CrOCl and CrOBr by computational methods including ab initio calculations, molecular dynamics, and Monte Carlo simulations.^[^ ^186^ ^]^ Bismuth oxyhalides, such as BiOBr nanoflakes, are discovered for their unique application in photonic devices, owing to their large nonlinear absorption coefficient β on the order of 10^−7^ m W^−1^ and a large Kerr coefficient n_2_ on the order of 10^−14^ m^2^ W^−1^.^[^ ^116d^ ^]^ BiOBr nanoflakes were integrated into 220‐nm‐thick silicon‐on‐insulator (SOI) waveguides, however, this device exhibited a larger propagation loss of ≈0.13 dB µm^−1^ than the reported values of graphene‐Si (0.07 dB µm^−1^)^[^ ^190^ ^]^ and MoS_2_‐Si waveguides (0.012 dB µm^−1^).^[^ ^191^ ^]^

## 5 Conclusion and Outlook

In this comprehensive review, we have conducted a detailed exploration of all families of layered anion‐mixed oxycompound materials, focusing on their crystal structures, preparation methods, properties, and potential applications. Layered anion‐mixed oxycompounds were classified into ternary, quaternary, and quinary compositions, incorporating additional anions such as chalcogenides, halides, or pnictogens beyond oxygen. Synthesis methods have evolved, encompassing both bottom‐up and top‐down approaches, enabling the fabrication of bulk, mono‐layered or few‐layered structures. Compared to compounds featuring single anion constituents, anion‐mixed oxycompounds exhibit distinctive optoelectronic, thermoelectric, magnetic, and ferroelectric properties, paving the way for diverse applications, including high‐efficiency photocatalysis, transistors, optoelectronic devices, and thermoelectric applications.

However, despite substantial advancements, several challenges persist, necessitating further investigation:

1. Scaling ultrathin 2D structures: For applications in electronic devices and catalysis, ultrathin 2D nanostructures are preferred. However, the majority of reported 2D oxycompounds exist predominantly in bulk form. Addressing this challenge requires the development of new methods to reliably produce large‐scale, high‐quality nanosheets of thinner 2D oxycompounds with multiple anions. Emerging strategies, such as self‐limited growth, offer promising avenues in this regard.^[^ ^192^ ^]^
2. Exploring novel materials: More attention should be directed toward novel materials that exhibit dynamic and energetic stability, such as monolayers of CrOCl, CrOBr, and CrOF.^[^ ^186^, ^193^ ^]^ Predicted through ab initio calculations to possess notable intrinsic ferromagnetic properties, these materials merit further investigation.
3. Expanding material diversity: While diverse 2D oxycompounds have been reported, there is still room to explore a broader range of such compounds. These possibilities can be pursued by altering the element's stoichiometry (Bi_2_O_2_S, Bi_4_O_4_S_3_, and Bi_9_O_7.5_S_6_) or introducing varying metallic cations (PbBiO_2_Cl and SrBiO_2_Cl, Li_2_LaTa_2_O_6_N and K_2_LaTa_2_O_6_N).
4. Correlating structure and performance: Establishing the correlation between material structure and performance is crucial for practical applications. Integrating theoretical computation, such as DFT, with experimentation can expedite the exploration of high‐performance 2D oxycompounds by predicting their structure and properties more efficiently.^[^ ^122^, ^194^ ^]^
5. Moving from laboratory findings to functional devices: While many layered anion‐mixed oxycompounds have been characterized for their fascinating properties, most remain at the materials characterization stage. It is imperative to apply these materials, particularly in 2D form, in devices to realize their practical applications.

Although substantial progress has been achieved in the research of layered anion‐mixed oxycompounds, further efforts to address remaining challenges are crucial to fully unlocking their potential and enabling their broader application in advanced technologies.

## Conflict of Interest

The authors declare no conflict of interest.
